# Inhibition of Wnt activity improves peri-implantation development of somatic cell nuclear transfer embryos

**DOI:** 10.1093/nsr/nwad173

**Published:** 2023-08-16

**Authors:** Yanhe Li, Caihong Zheng, Yingdong Liu, Jincan He, Qiang Zhang, Yalin Zhang, Xiaochen Kou, Yanhong Zhao, Kuisheng Liu, Dandan Bai, Yanping Jia, Xiaoxiao Han, Yifan Sheng, Jiqing Yin, Hong Wang, Shuai Gao, Wenqiang Liu, Shaorong Gao

**Affiliations:** Shanghai Key Laboratory of Maternal Fetal Medicine, Clinical and Translational Research Center of Shanghai First Maternity and Infant Hospital, Shanghai Institute of Maternal-Fetal Medicine and Gynecologic Oncology, Shanghai Key Laboratory of Signaling and Disease Research, Frontier Science Center for Stem Cell Research, School of Life Sciences and Technology, Tongji University, Shanghai 200092, China; CAS Key Laboratory of Genomic and Precision Medicine, Beijing Institute of Genomics, Chinese Academy of Sciences and China National Center for Bioinformation, Beijing 100101, China; Shanghai Key Laboratory of Maternal Fetal Medicine, Clinical and Translational Research Center of Shanghai First Maternity and Infant Hospital, Shanghai Institute of Maternal-Fetal Medicine and Gynecologic Oncology, Shanghai Key Laboratory of Signaling and Disease Research, Frontier Science Center for Stem Cell Research, School of Life Sciences and Technology, Tongji University, Shanghai 200092, China; Shanghai Key Laboratory of Maternal Fetal Medicine, Clinical and Translational Research Center of Shanghai First Maternity and Infant Hospital, Shanghai Institute of Maternal-Fetal Medicine and Gynecologic Oncology, Shanghai Key Laboratory of Signaling and Disease Research, Frontier Science Center for Stem Cell Research, School of Life Sciences and Technology, Tongji University, Shanghai 200092, China; Key Laboratory of Animal Genetics, Breeding and Reproduction of the MARA, National Engineering Laboratory for Animal Breeding, College of Animal Science and Technology, China Agricultural University, Beijing 100193, China; Shanghai Key Laboratory of Maternal Fetal Medicine, Clinical and Translational Research Center of Shanghai First Maternity and Infant Hospital, Shanghai Institute of Maternal-Fetal Medicine and Gynecologic Oncology, Shanghai Key Laboratory of Signaling and Disease Research, Frontier Science Center for Stem Cell Research, School of Life Sciences and Technology, Tongji University, Shanghai 200092, China; Shanghai Key Laboratory of Maternal Fetal Medicine, Clinical and Translational Research Center of Shanghai First Maternity and Infant Hospital, Shanghai Institute of Maternal-Fetal Medicine and Gynecologic Oncology, Shanghai Key Laboratory of Signaling and Disease Research, Frontier Science Center for Stem Cell Research, School of Life Sciences and Technology, Tongji University, Shanghai 200092, China; Shanghai Key Laboratory of Maternal Fetal Medicine, Clinical and Translational Research Center of Shanghai First Maternity and Infant Hospital, Shanghai Institute of Maternal-Fetal Medicine and Gynecologic Oncology, Shanghai Key Laboratory of Signaling and Disease Research, Frontier Science Center for Stem Cell Research, School of Life Sciences and Technology, Tongji University, Shanghai 200092, China; Shanghai Key Laboratory of Maternal Fetal Medicine, Clinical and Translational Research Center of Shanghai First Maternity and Infant Hospital, Shanghai Institute of Maternal-Fetal Medicine and Gynecologic Oncology, Shanghai Key Laboratory of Signaling and Disease Research, Frontier Science Center for Stem Cell Research, School of Life Sciences and Technology, Tongji University, Shanghai 200092, China; Shanghai Key Laboratory of Maternal Fetal Medicine, Clinical and Translational Research Center of Shanghai First Maternity and Infant Hospital, Shanghai Institute of Maternal-Fetal Medicine and Gynecologic Oncology, Shanghai Key Laboratory of Signaling and Disease Research, Frontier Science Center for Stem Cell Research, School of Life Sciences and Technology, Tongji University, Shanghai 200092, China; Shanghai Key Laboratory of Maternal Fetal Medicine, Clinical and Translational Research Center of Shanghai First Maternity and Infant Hospital, Shanghai Institute of Maternal-Fetal Medicine and Gynecologic Oncology, Shanghai Key Laboratory of Signaling and Disease Research, Frontier Science Center for Stem Cell Research, School of Life Sciences and Technology, Tongji University, Shanghai 200092, China; Shanghai Key Laboratory of Maternal Fetal Medicine, Clinical and Translational Research Center of Shanghai First Maternity and Infant Hospital, Shanghai Institute of Maternal-Fetal Medicine and Gynecologic Oncology, Shanghai Key Laboratory of Signaling and Disease Research, Frontier Science Center for Stem Cell Research, School of Life Sciences and Technology, Tongji University, Shanghai 200092, China; Shanghai Key Laboratory of Maternal Fetal Medicine, Clinical and Translational Research Center of Shanghai First Maternity and Infant Hospital, Shanghai Institute of Maternal-Fetal Medicine and Gynecologic Oncology, Shanghai Key Laboratory of Signaling and Disease Research, Frontier Science Center for Stem Cell Research, School of Life Sciences and Technology, Tongji University, Shanghai 200092, China; Shanghai Key Laboratory of Maternal Fetal Medicine, Clinical and Translational Research Center of Shanghai First Maternity and Infant Hospital, Shanghai Institute of Maternal-Fetal Medicine and Gynecologic Oncology, Shanghai Key Laboratory of Signaling and Disease Research, Frontier Science Center for Stem Cell Research, School of Life Sciences and Technology, Tongji University, Shanghai 200092, China; Shanghai Key Laboratory of Maternal Fetal Medicine, Clinical and Translational Research Center of Shanghai First Maternity and Infant Hospital, Shanghai Institute of Maternal-Fetal Medicine and Gynecologic Oncology, Shanghai Key Laboratory of Signaling and Disease Research, Frontier Science Center for Stem Cell Research, School of Life Sciences and Technology, Tongji University, Shanghai 200092, China; Key Laboratory of Animal Genetics, Breeding and Reproduction of the MARA, National Engineering Laboratory for Animal Breeding, College of Animal Science and Technology, China Agricultural University, Beijing 100193, China; Shanghai Key Laboratory of Maternal Fetal Medicine, Clinical and Translational Research Center of Shanghai First Maternity and Infant Hospital, Shanghai Institute of Maternal-Fetal Medicine and Gynecologic Oncology, Shanghai Key Laboratory of Signaling and Disease Research, Frontier Science Center for Stem Cell Research, School of Life Sciences and Technology, Tongji University, Shanghai 200092, China; Shanghai Key Laboratory of Maternal Fetal Medicine, Clinical and Translational Research Center of Shanghai First Maternity and Infant Hospital, Shanghai Institute of Maternal-Fetal Medicine and Gynecologic Oncology, Shanghai Key Laboratory of Signaling and Disease Research, Frontier Science Center for Stem Cell Research, School of Life Sciences and Technology, Tongji University, Shanghai 200092, China

**Keywords:** SCNT, reprogramming, implantation, Wnt, naïve pluripotency, primed pluripotency, epiblast, single-cell RNA-seq, epigenetic barrier

## Abstract

Somatic cell nuclear transfer (SCNT) can reprogram differentiated somatic cells into totipotency. Although pre-implantation development of SCNT embryos has greatly improved, most SCNT blastocysts are still arrested at the peri-implantation stage, and the underlying mechanism remains elusive. Here, we develop a 3D *in vitro* culture system for SCNT peri-implantation embryos and discover that persistent Wnt signals block the naïve-to-primed pluripotency transition of epiblasts with aberrant H3K27me3 occupancy, which in turn leads to defects in epiblast transformation events and subsequent implantation failure. Strikingly, manipulating Wnt signals can attenuate the pluripotency transition and H3K27me3 deposition defects in epiblasts and achieve up to a 9-fold increase in cloning efficiency. Finally, single-cell RNA-seq analysis reveals that Wnt inhibition markedly enhances the lineage developmental trajectories of SCNT blastocysts during peri-implantation development. Overall, these findings reveal diminished potentials of SCNT blastocysts for lineage specification and validate a critical peri-implantation barrier for SCNT embryos.

## INTRODUCTION

Somatic cell nuclear transfer (SCNT) has unique abilities to reprogram the differentiated somatic cell into a totipotent state and produce a viable cloned animal [1–3], and has great potential in animal reproductive and regenerative medicine. However, despite the successful application of this approach to more than 20 species, cloning efficiency remains extremely low, which is commonly manifested in developmental arrest prior to implantation and abnormal fetal and placental development during the post-implantation period [4]. Recent progress has been made in identifying critical epigenetic barriers and overcoming these abnormalities to improve cloning efficiency, and these improvements have largely contributed to the success of cloning monkeys [5,6]. Correction of abnormal histone modifications, DNA re-methylation and ectopic *Xist* expression in the pre-implantation stage could significantly improve SCNT embryo development [7–12]. Recovering aberrant H3K27me3-dependent imprinted gene expression could prevent placental overgrowth and post-implantation development [10,13–15]. However, the cloning efficiency of these improved approaches is still low compared with that of naturally fertilized or IVF embryos.

Indeed, it is worth noting that most SCNT embryos reaching the blastocyst stage are still arrested at the peri-implantation period of gestation, which indicates the existence of other severe barriers preventing SCNT embryo development at this stage [16–18]. However, SCNT blastocysts invading the uterine stroma are hidden from view, and critical events, such as dramatic morphogenetic transformations and cell fate decisions of multiple lineages, remain mysterious. Compared with our broader knowledge of the molecular mechanisms that occur during pre- and post-implantation development, the peri-implantation stages of SCNT embryos have been poorly investigated due to the difficulties with accessing the embryos.

Notably, the *in vitro* culture systems recently developed for mimicking peri- and post-implantation development provide important information to better understand the molecular and morphological events of the perigastrulation stage [19–22]. Recently, Ma *et al.* reported an optimized 2D system with Matrigel-coated glass-bottomed dishes that can promote egg cylinder formation, and demonstrated that cells from these *in vitro* cultured embryos are similar to their *in vivo* counterparts [23]. Thus, approaches that combine the optimized 3D *in vitro* culture system with multiomics analysis have the potential to reveal critical barriers to the peri-implantation development of somatic cell cloned embryos.

## RESULTS

### Disorganized epiblast of SCNT peri-implantation embryos cultured *in vitro*

**Figure 1.**
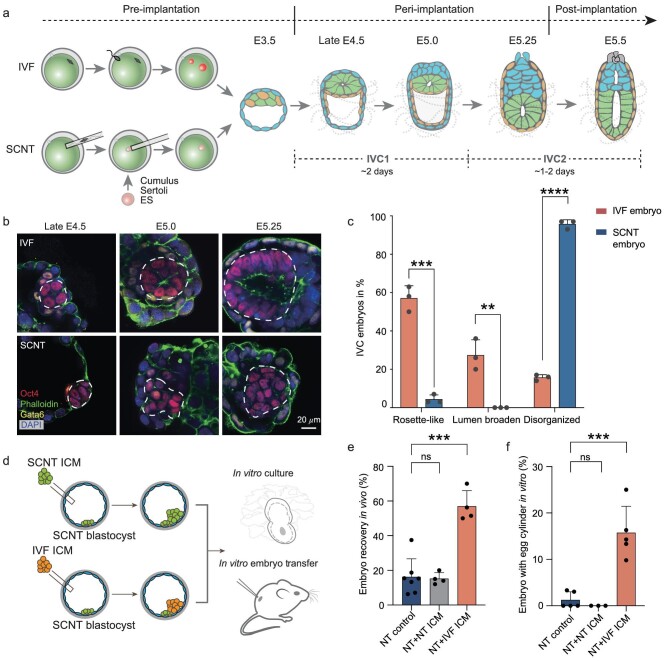
Disorganized EPI development of SCNT peri-implantation embryos. (a) Schematic representation of IVF and SCNT peri-implantation embryo generation in a 3D IVC system. (b) Morphology of the EPI structures in IVF (top) and SCNT (bottom) embryos at late E4.5, E5.0 and E5.25. Embryos were co-stained for *Oct4, Gata6, Par6* and DAPI. The EPI is encircled by a dotted white line. Scale bar, 20 μm. (c) Percentage of rosette or lumen structure formation in *in vitro* cultured IVF and SCNT embryos at E5.25. (d) Schematic representation of the IVF-ICM-cell and NT-ICM-cell complementation assays. (e) Percentage of *in vivo* embryo recovery (E7.5) in the SCNT embryos complemented with IVF and SCNT ICM cells. (f) Percentage of IVC embryos that developed into egg cylinders (E5.5) in SCNT embryos complemented with IVF and SCNT ICM cells. For (c), (e) and (f), *P*-values were determined using an unpaired two-tailed *t*-test; error bars and means ± SD are shown for *n* ≥ 3 experiments.

To uncover the black box of peri-implantation development of SCNT embryos, we developed a Matrigel-packed 3D *in vitro* culture (IVC) system for supporting the suspension culture of peri-implantation mouse IVF and SCNT embryos embedding in 25% Matrigel in IVC medium (Fig. 1a). Similar to *in vivo* mouse embryos, the *in vitro* cultured IVF embryos in the 3D platform underwent dynamic transformation between E4.5 and E5.5 (Supplementary Fig. S1a, b and e). In principle, upon implantation, the epiblast (EPI) of the blastocyst acquires epithelial polarity and forms a highly organized rosette-like structure (late E4.5) [24]. The lumen subsequently appears in the center of the rosette (E5.25) (Fig. 1b). This process is called EPI transformation [20]. However, we found that very few cumulus cell-derived SCNT embryos underwent normal transformation during peri-implantation development under the same condition (Fig. 1c and Supplementary Fig. S1c and d). Compared with IVF embryos (84.3 ± 1.5%) that formed rosette or lumen structures, only 4.3 ± 2.3% of the SCNT embryos were polarized into a rosette-like structure, and their EPI cells remained underdeveloped and poorly organized (Fig. 1c).

After 4 days of culture, 40.4 ± 14.7% of IVF embryos developed to the point of emerging the egg cylinder (E5.5) while <3% of SCNT embryos developed normally, with the majority arrested at E4.5–E5.0 (Supplementary Fig. S1f–h). Detailed morphological and immunofluorescent staining revealed that the EPI development of SCNT embryos was seriously aberrant, which mainly manifested as a reduction in the number of pluripotent EPI cells at late E4.5 and then an amorphous cell shape at E5.25 (Fig. 1b and Supplementary Fig. S1d). Similarly, we observed the same morphological abnormalities in cloned embryos when Sertoli cells and embryonic stem cells (ESCs) were used as donor cells (Supplementary Fig. S2a and b). Together, these results suggested that the rosette-stage organization of the EPI was aberrant in the peri-implantation SCNT embryos.

To test whether the quality or the cell number of EPI in SCNT embryos could lead to the abnormal development of SCNT embryos during the peri-implantation period, we performed a blastocyst complementation assay (Fig. 1d). Eight to ten inner cell mass (ICM) cells isolated from IVF or SCNT embryos were injected into pre-implantation SCNT blastocysts. Then, we subjected these blastocysts to *in vitro* culture and *in vivo* transfer. Remarkably, the SCNT embryos injected with IVF ICM cells displayed a much higher *in vitro* egg cylinder formation rate and *in vivo* embryo recovery rate than the controls and SCNT embryos injected with SCNT ICM cells (Fig. 1e, f and Supplementary Fig. S2c and d). Immunofluorescence staining analysis of IVF ICM cell-complemented SCNT embryos showed normal lumen structures of EPI (Supplementary Fig. S2e). Therefore, these results suggested that the quality, rather than the cell number, of EPI determines the rosette-like structure formation and subsequent embryo development.

### Aberrant naïve-to-primed pluripotency transition of EPI during peri-implantation SCNT embryo development

**Figure 2.**
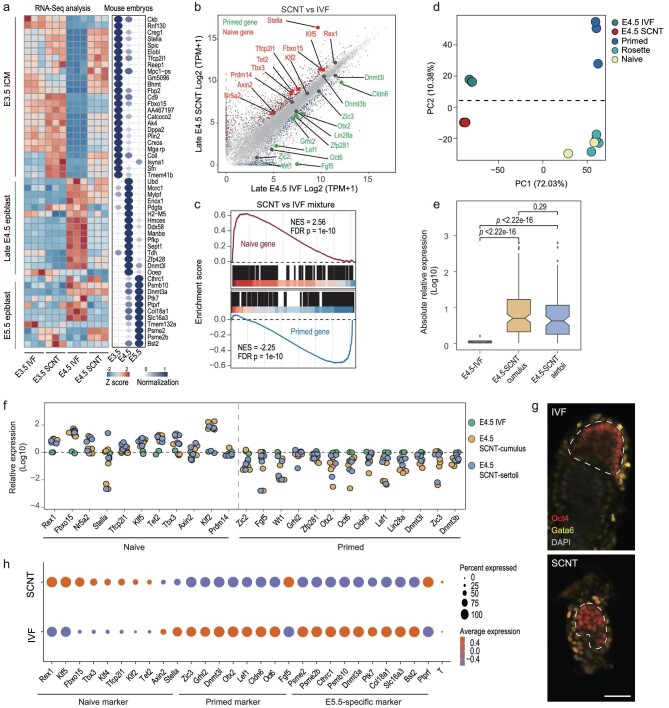
Aberrant naïve-to-primed pluripotency transition of EPI during peri-implantation SCNT embryo development. (a) Comparison of lineage-specific gene expression among E3.5 ICM and late-E4.5 EPI of IVF and SCNT embryos. (b) Comparison of gene expression profiles of the late-E4.5 EPI of IVF and SCNT embryos. (c) Gene set enrichment analysis (GSEA) of specific genes expressed in naïve and primed ESCs in the late-E4.5 EPI of IVF and SCNT embryos. NES, normalized enrichment score. (d) PCA comparison of gene expression profiles among the late-E4.5 EPI of IVF and SCNT embryos, naïve ESCs, primed ESCs and rosette-like cells (RSCs). Published transcriptome data of stem cell lines were obtained from GSE145727. (e) Absolute value comparison of the relative expression of markers in (e) to late-E4.5 IVF EPI between groups of IVF, cumulus and Sertoli cell-derived SCNT embryos. *P*-values were determined using the Wilcoxon signed-rank test. (f) Expression of pluripotency markers on the late-E4.5 EPI of single IVF embryos and SCNT embryos derived from cumulus and Sertoli cells. (g) Confocal images of E5.5 IVF and SCNT embryos *in vivo*, co-stained for *Oct4, Gata6* and DAPI. Scale bar, 50 μm. (h) Gene expression levels and variability of naïve, primed, E5.5-specific and mesenchymal markers in scRNA-seq of E5.5 IVF and SCNT embryos *in vivo*.

To identify the molecular characteristics of the disorganization of EPI in peri-implantation SCNT embryos, we isolated EPI cells from primitive endoderm (PrE) and Trophoblast cells using a micromanipulator (Supplementary Fig. S3a and b), and performed RNA-seq for the E3.5 ICM and late-E4.5 EPI of IVF and SCNT embryos. A comparison of the transcriptomes showed distinct expression patterns between these embryos during the peri-implantation process (Supplementary Fig. S3c and d). Furthermore, we compared our data with public single-cell RNA-seq (scRNA-seq) data sets of *in vivo* mouse embryos [25]. Unlike the correct transcriptional profiles of lineage-specific genes and cell fate conversion of the IVF groups at E3.5 to late E4.5 (Fig. 2a), the transcriptional profiles were compromised in SCNT embryos. The naïve-to-primed pluripotency transition is a pivotal event in lineage specification and peri-implantation embryo development [26]. Intriguingly, a significant proportion of naïve genes, such as *Tet2, Rex1* and *Stella*, were generally maintained, whereas the primed genes, such as *Otx2, Oct6* and *Fgf5*, were generally depressed in the EPI of SCNT embryos compared with that of IVF embryos around E3.5 to late E4.5 (Fig. 2b and Supplementary Fig. S3e). Quantitative PCR with reverse transcription (RT-qPCR) analysis of several representative naïve and primed genes also showed the same expression pattern (Supplementary Fig. S3f).

Gene set enrichment analysis (GSEA) using the top 200 naïve specific-upregulated genes and the top 200 primed specific-upregulated genes between naïve and primed ESCs from a published study further confirmed that the EPI of SCNT embryos was enriched with naïve pluripotency, while the EPI of IVF embryos was enriched with primed pluripotency (Fig. 2c) [27]. We further performed single-embryo RNA-seq analysis on the EPI of IVF and cumulus or Sertoli cell-derived SCNT embryos to exclude variation and heterogeneity. Indeed, the differential expression patterns of pluripotent genes between them resembled those of the mixed samples (Fig. 2d–f, and Supplementary Fig. S3g and h).

Pluripotency is highly dynamic in the pre- to post-implantation mammalian embryo. Multiple types of pluripotency stem cells, including naïve, formative and primed cells, recapitulate EPI lineage progress *in vivo* [27–30]. Principle component analysis (PCA) of these public data combined with our in-house data showed that the developmental progression could be captured in PC2 (Fig. 2d). As expected, the E4.5 ICM of cumulus or Sertoli cell-derived SCNT embryos mapped was close to that of the naïve ESCs, while the E4.5 ICM in IVF embryos showed greater similarity with primed ESCs along this developmental axis (Fig. 2d and Supplementary Fig. S3i).

To further examine the pluripotency state of the EPI of SCNT and IVF embryos *in vivo*, we performed scRNA-seq on them. Morphological examination of the SCNT embryos (E5.5) *in vivo* also revealed a poorly organized EPI structure (Fig. 2g). Consistent with our *in vitro* experiments, the pluripotency-marker and lineage-specific genes displayed a more naïve pluripotent state in the EPI cells of SCNT embryos than IVF embryos (Fig. 2h), which indicated that the EPI of *in vivo* E5.5 SCNT embryos was also stuck in the naïve state and strongly supported our *in vitro* data.

In sum, these results suggested that the naïve-to-primed pluripotency transition of EPI is blocked following SCNT embryo implantation, which is closely linked to defects in EPI transformation events and unveils the presence of barriers that prevent this transition.

### Abnormal H3K27me3 remodeling during peri-implantation SCNT embryo development

Pervasive epigenetic reprogramming and transcriptional regulation changes from naïve EPI (E3.5) toward a primed state (E6.5) have been shown to orchestrate an intricate interaction network for promoting peri-implantation development [31,32]. Aberrant transcriptome transitions of SCNT embryos prompted us to investigate epigenetic reprogramming events during this process.

**Figure 3.**
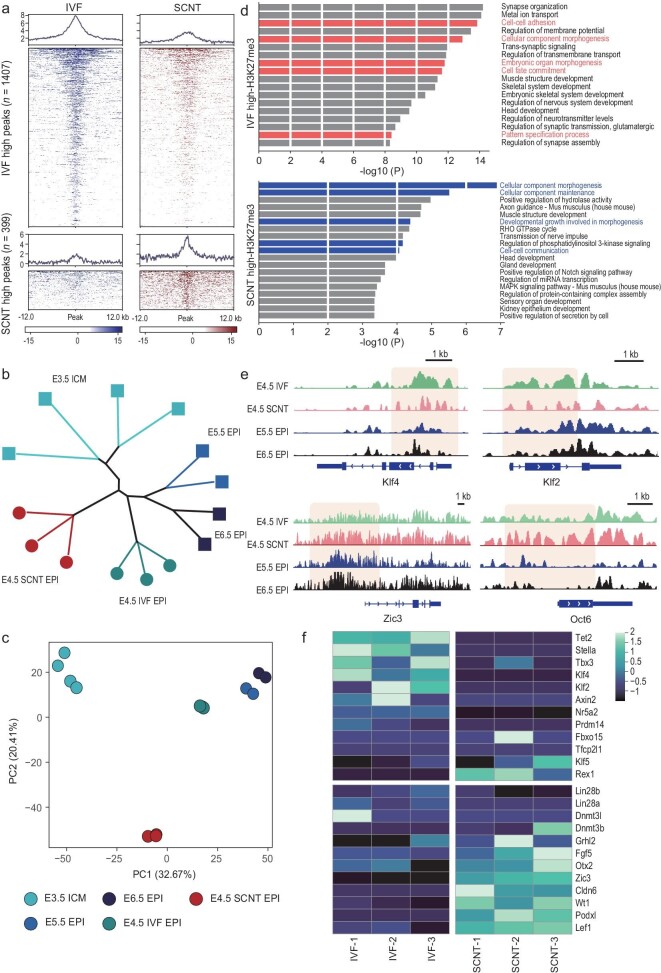
Abnormal H3K27me3 remodeling during peri-implantation SCNT embryo development. (a) Heat map centered over promoters of genes with differential deposition of H3K27me3 between the late-E4.5 EPI of IVF and SCNT embryos. (b) Clustering dendrogram of the late-E4.5 EPI of IVF and SCNT embryos, and E3.5 ICM to E6.5 EPI based on H3K27me3 peak signals. Published H3K27me3 ChIP-seq data of EPI cells were obtained from GSE76687. (c) PCA plot of the late-E4.5 EPI of IVF and SCNT embryos, and E3.5 ICM to E6.5 EPI based on the H3K27me3 levels. Published H3K27me3 ChIP-seq data of EPI cells were obtained from GSE76687. (d) GO analysis of differential H3K27me3 enriched genes in late-E4.5 EPI of SCNT embryos compared with that of IVF embryos. (e) Integrative Genomics Viewer (IGV) snapshots showing levels of H3K27me3 signals of *Klf4* and *Klf2* (naïve marker) and *Oct6* and *Zic3* (primed marker). Regions showing differential H3K27me3 around promoter regions between E4.5 IVF and SCNT EPI are shaded. (f) Heat map showing H3K27me3 levels for the naïve and primed markers between late-E4.5 EPI of IVF and SCNT embryos.

Extensive H3K27me3 modifications are gradually reallocated to promoter regions in post-implantation embryos [33]. H3K27me3 deficiency leads to embryonic arrest around gastrulation. Therefore, to examine whether H3K27me3 reprogramming is involved in the regulation of pluripotency transitions among the SCNT peri-implantation embryos, we next performed ultra-low-input microcaoccal nuclease-based native ChIP (ULI-NChIP)-seq for H3K27me3 in the late-E4.5 EPI of IVF and SCNT embryos. We could observe that H3K27me3 signals were restored at the promoters of developmental genes following IVF and SCNT embryonic implantation (Supplementary Fig. S4a). Intriguingly, unlike the similar distribution patterns at morula stage between IVF and SCNT embryos [10], the global H3K27me3 level was obviously distinct between their EPI at late-E4.5 stage (Fig. 3a and Supplementary Fig. S4b and c). These regions of differentially expressed genes are where the enrichment of H3K27me3 was largely compromised (Supplementary Fig. S4d). ChIP-seq-based PCA combined with the public ChIP-seq data sets (E3.5–E6.5 EPI) revealed that late-E4.5 IVF EPI was close to the development trajectory [30], but late-E4.5 SCNT EPI obviously deviated from this path. The hierarchical clustering analysis further supported the PCA results (Fig. 3b and c). These results demonstrate that an aberrant global H3K27me3 pattern is established among these EPI cells shortly after SCNT blastocyst implants. Gene ontology (GO) analyses revealed that these differential H3K27me3-enriched regions among IVF and SCNT groups were involved in epithelial cell proliferation and morphogenesis, cell–cell adhesion and the pattern specification process, which is strongly related to the phenotypic abnormalities observed in the EPI of SCNT embryos (Fig. 3d). More importantly, in contrast to the IVF groups, the majority of naïve markers upregulated were accompanied by lower H3K27me3, whereas primed markers were downregulated with retained H3K27me3 signals among the SCNT groups (Fig. 3e and f, and Supplementary Fig. S4e), suggesting that the aberrant H3K27me3 reprogramming of EPI in SCNT peri-implantation embryos might impede the naïve-to-primed pluripotency transition program.

### Wnt activity persists upon implantation of SCNT embryos

**Figure 4.**
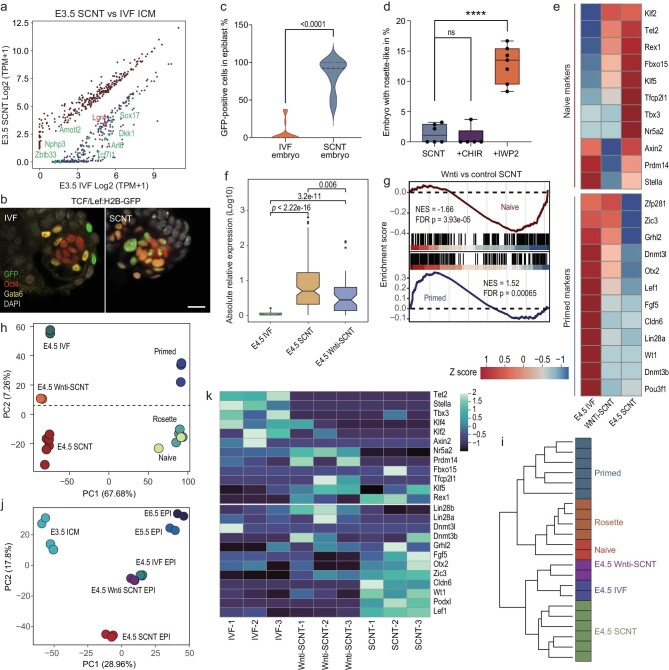
Wnt inhibition greatly improves the development of SCNT embryos. (a) Comparison of Wnt-related genes in differential gene expression profiles of the ICM of IVF and SCNT blastocysts. The genes marked are Wnt antagonists or activators. (b) TCF/Lef: H2B-GFP IVF (left) and SCNT (right) embryos at late E4.5 stained for GFP, *Oct4, Gata6* and DAPI. Scale bar, 20 μm. (c) Percentage of GFP-positive cells in the EPI of IVF and SCNT embryos. (d) Percentage of rosette-like SCNT embryos generated with no treatment, Wnt activator CHIR treatment and Wnt inhibitor IWP2 treatment. (e) Comparison of pluripotent marker gene expression among the IVF-, SCNT- and Wnti-treated SCNT embryos. (f) Absolute value comparison of the relative expression of markers in (d) to late-E4.5 IVF EPI between groups of IVF-, SCNT- and Wnti-SCNT embryos. *P*-values were determined using the Wilcoxon signed-rank test. (g) GSEA of specific genes expressed in naïve and primed ESCs between the late-E4.5 EPI of untreated and Wnti-SCNT embryos. NES, normalized enrichment score. (h) PCA comparison of gene expression profiles among the late-E4.5 EPI of IVF-, SCNT- and Wnti-SCNT embryos, naïve ESCs, primed ESCs and RSCs. Published transcriptome data of stem cell lines were obtained from GSE145727. (i) Hierarchical clustering analysis of the late-E4.5 EPI of IVF, untreated and Wnti-SCNT embryos, naïve ESCs, primed ESCs and RSCs. (j) PCA plot of E3.5 ICM, E5.5 EPI, E6.5 EPI, late-E4.5 EPI of IVF, SCNT and Wnti-SCNT embryos based on the H3K27me3 levels. (k) Heat map showing the H3K27me3 levels for the naïve and primed markers between late-E4.5 EPI of IVF, SCNT and Wnti-SCNT embryos. For (c) and (d), *P*-values were determined using the unpaired two-tailed *t*-test; error bars and means ± SD are shown for *n* ≥ 3 experiments.

To further dissect the molecular mechanism responsible for this aberrant naïve-to-primed pluripotency transition in SCNT embryos, we first conducted a transcriptome analysis of the E3.5 and late-E4.5 SCNT embryos, in which disorganization of EPI was initiated. GO analysis of SCNT embryo changes revealed enrichment in several categories compared with that of the IVF control (Supplementary Fig. S5a). Among them, the activation of the Wnt signaling pathway in these two stages might point to EPI cell morphogenesis, which was also reported to be indispensable for the regulation of pluripotency transitions [34]. By a careful analysis of differential transcriptome data between the ICM cells of E3.5 IVF and SCNT embryos (Fig. 4a), we found that eight Wnt-related genes were significantly differentially expressed, among which seven well-known Wnt signaling antagonists, such as *Dkk1, Sox17* and *Tcf7l1*, were significantly downregulated in SCNT groups. In line with this, *Otx2*, as a precursor effector of the Wnt pathway, was lacking in late-E4.5 SCNT embryos (Supplementary Fig. S5b). Taken together, these data indicate persistent abnormal Wnt signaling activity in SCNT embryos.

To further corroborate this presumption, we injected the piggyBac transposon-mediated TCF/Lef: H2B-GFP reporter system into SCNT and IVF embryos to directly monitor canonical Wnt pathway activity at single-cell resolution (Supplementary Fig. S5c). Indeed, we observed more robust Wnt reporter activity in SCNT late blastocysts (Supplementary Fig. S5d and e). Following implantation, the IVF embryos displayed few EPI cells with Wnt reporter activity, consistent with a recent report [35], whereas the SCNT embryos displayed Wnt reporter activity in the majority of EPI cells at the late-E4.5 stage, suggesting abnormal activation of the Wnt pathway in peri-implantation SCNT embryos (Fig. 4b and c). To mimic the persistent Wnt signaling of SCNT peri-implantation embryos, we treated IVF embryos with the Wnt activator CHIR99021 (CHIR) at E3.0–E4.5 (Supplementary Fig. S6a). Intriguingly, the normal organization of rosette-like structure *in vitro* was also blocked, accompanied by robust Wnt reporter activity in the EPI cells (Supplementary Fig. S6b). Moreover, the expression of pluripotency markers of the CHIR-treated IVF and SCNT embryos was examined. Compared with the IVF control group, the naïve markers of CHIR-treated IVF and SCNT embryos were all maintained and primed markers were obviously depressed (Supplementary Fig. S6c). These phenotypes were highly similar to what were found in the EPI of SCNT embryos (Supplementary Fig. S6d), suggesting that persistent activation of Wnt signaling might be a major cause of the peri-implantation failure of SCNT embryos.

The above observation prompted us to hypothesize that the critical factor of the persistent activation of Wnt signaling is the aberrant epigenetic reprogramming in donor somatic cells. Combining published histone modification data sets of mouse somatic cells and early embryos, we found that H3K27me3 [10,36], but not other modifications, was strongly enriched at the promoter of *Dkk1* in donor somatic cells and still maintained in the SCNT morulae, while no obvious enrichment was observed in the normal morulae (Supplementary Fig. S6e). These results suggested that aberrant epigenetic reprogramming at the important Wnt signaling factors had already existed in the early stage of SCNT embryos.

### Inhibition of Wnt drives naïve-to-primed transition in the EPI of SCNT embryos

Having established a correlation between the abnormal activation of Wnt signaling and the peri-implantation development failure of SCNT embryos, we next aimed to test whether the inhibition of persistent Wnt signals can promote naïve-to-primed pluripotency transition and rosette-like structure formation in SCNT embryos. We treated SCNT embryos with the Wnt inhibitor (Wnti) IWP2 at different time points during the pre- or peri-implantation stage (Supplementary Fig. S6f). Surprisingly, we found that the rosette formation efficiency at E5.0 increased from 1.3 ± 1.5% to 12.8 ± 3.0% upon treatment with IWP2 at E3.0–E4.5 but remained unchanged with IWP2 treatment at E1.5–E3.0 and E1.5–E4.5 and with Wnt activator CHIR treatment (Fig. 4d and Supplementary Fig. S6g–i).

To characterize the underlying molecular features in detail, RNA-seq was performed for EPI cells of Wnti treated SCNT (Wnti-SCNT) embryos at the late-E4.5 stage. Transcriptome analysis showed that many active naïve markers, such as *Tet2, Rex1* and *Axin2*, were obviously depressed compared with the EPI of untreated SCNT embryos, whereas downregulated primed markers, such as *Otx2, Dnmt3l* and *Zfp281*, were upregulated (Fig. 4e and f). The GSEA results further confirmed that the EPI of Wnti-SCNT embryos was enriched with primed genes, while the EPI of untreated SCNT embryos was enriched with naïve genes (Fig. 4g). A comparison of the EPI of Wnti-SCNT embryos and IVF embryos was performed by PCA, and the results revealed that the EPI of Wnti-SCNT samples mapped alongside that of the IVF samples but was separate from that of the untreated SCNT samples (Fig. 4h). A clustering analysis also revealed that the EPIs of Wnti-SCNT samples were clustered together with those of IVF samples (Fig. 4i). Previous studies have shown that inhibition of Wnt signaling promotes H3K27me3 remodeling and naïve-to-primed state transitions of embryonic stem cells [27,37]. Therefore, we performed ChIP-seq analysis and observed that the aberrant H3K27me3-enriched regions in late-E4.5 EPI of SCNT embryos were significantly rescued in the Wnti-SCNT embryos (Supplementary Fig. S4b and c). GO analysis of these rescued regions revealed processes involving embryonic morphogenesis, cell differentiation and regulation of embryonic development (Supplementary Fig. S4f). ChIP-seq-based PCA and clustering analysis revealed that the EPI of Wnti-SCNT samples was obviously close to that of the IVF samples (Fig. 4j and Supplementary Fig. S4g). Moreover, we found that aberrant H3K27me3 patterns at naïve and primed gene sets were fully or partially restored in these EPI cells of Wnti-SCNT embryos (Fig. 4k and Supplementary Fig. S4e), indicating that Wnt signal changes could regulate H3K27me3 depositions upon these pluripotency factors and further drive pluripotency transitions of EPI in peri-implantation SCNT embryos.

### Wnt inhibition greatly improves the development of SCNT embryos

To examine whether manipulating Wnt inhibition could promote *in vivo* development and implantation of SCNT embryos, the Wnt inhibitors IWP2 and IWR-1-endo were used alone or in combination during E3.0–E4.0 of the pre-implantation stage. We first manipulated IVF embryos with Wnti and transferred them into the uteri of pseudo-pregnant mice. Compared with the untreated embryos, the implantation and embryo recovery rates showed no difference with Wnti treatment (Supplementary Fig. S7a and b), suggesting no toxic effect of Wnti on the *in vivo* development of normal embryos. We then treated SCNT embryos with Wnti at E3.0–4.0 and performed a blastocyst transfer to assess *in vivo* developmental ability. Excitingly, the Wnti-SCNT embryos at the E7.5 stage that were recovered from implantation sites were significantly improved and displayed normal embryonic morphology compared with those of the untreated SCNT embryos (Supplementary Fig. S7c and d, Table S1).

**Figure 5.**
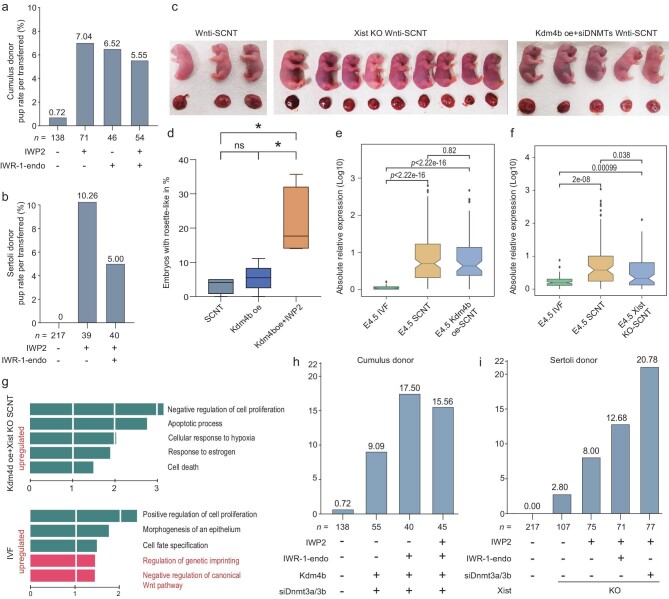
Combined Wnt inhibition with other epigenetic correction treatments further improved cloning efficiency. (a and b) Pup rate of SCNT embryos treated with Wnti alone. SCNT embryos derived from cumulus cells (a) and Sertoli cells (b) treated with Wnt inhibitor. (c) Generation of fetuses and placentas from singular approaches (cumulus-derived SCNT embryos treated with Wnti) and combined approaches (*Kdm4b*oe + si*Dnmt3a/3b* + Wnti treatment or *Xist* KO Sertoli cell-derived SCNT embryos treated with Wnti). (d) Percentage of rosette-like-structure formation at E5.0 in *Kdm4b*-injection SCNT embryos with Wnti and no treatment. (e) Absolute value comparison of the relative expression of pluripotency markers in the late-E4.5 IVF EPI between groups of IVF-, SCNT- and *Kdm4b*-injection SCNT embryos. *P*-values were determined using the Wilcoxon signed-rank test. (f) Absolute value comparison of the relative expression of pluripotency markers in the late-E4.5 IVF EPI between groups of IVF-, SCNT- and *Xist* KO donor SCNT embryos. *P*-values were determined using Wilcoxon signed-rank test. (g) The enrichment of GO terms of up- and downregulated genes between the IVF and Kdm4doe + Xist KO donor SCNT embryos. The *P-*values were calculated based on a hypergeometric test using Metascape. Published transcriptome data were obtained from GSE214878. (h) Pup rate of SCNT embryos with the combined (*Kdm4b*oe + si*Dnmt3a/3b* + Wnti) approach. (i) Pup rate of SCNT embryos using Xist KO Sertoli donor cells with the combined (si*Dnmt3a/3b* + Wnti) approach. For (d), *P*-values were determined using the unpaired two-tailed t-test; error bars and means  ± SD are shown for n ≥ 3 experiments.

To identify whether the Wnti treatment could improve the survival rate of SCNT embryos, we allowed embryos to complete full-term development. When cumulus cells were used as donor cells, only 0.72% of transferred blastocysts in the SCNT control reached term, and the result was even lower for 2-cell transferred embryos, which is consistent with previous reports [38,39], suggesting the negative impact of *in vitro* culture on SCNT pre-implantation embryo development. Nonetheless, 5.55%–7.04% (up to a 9-fold increase) of transferred Wnti-SCNT blastocysts developed to term (Fig. 5a). Similar experiments were also performed using Sertoli cells as donor cells, and up to 10.3% of transferred Wnti-SCNT blastocysts developed to term (Fig. 5b and c), which was significantly higher than the SCNT control (0%). Thus, these results indicate that overcoming the implantation barrier of SCNT embryos using Wnt inhibitors could significantly improve the full-term development rate. Moreover, we observed that the weight and spongiotrophoblast layers of the Wnti-SCNT placentae were partly improved (Supplementary Fig. S7e), indicating that promotion of the pluripotency transition in EPI lineage of SCNT embryos might be beneficial for extra-embryonic development.

### Wnt inhibition combined with other epigenetic correction approaches further improves cloning efficiency

Removing somatic H3K9me3 could also significantly improve the developmental potential of SCNT embryos [6,8,9,40]. To verify whether abnormal Wnt activation still exists in H3K9me3 demethylation treatment embryos, we removed somatic H3K9me3 by *Kdm4b* mRNA injection, and the blastocyst rate increased from 40% to 88%, which was consistent with our previous reports (Supplementary Fig. S7f) [9]. We cultured *Kdm4b*-injection blastocysts for peri-implantation observation, and the results showed that 94.48% of *Kdm4b*-injected SCNT embryos still failed to form rosette-like structures at the E5.25 stage and were accompanied by robust Wnt reporter activity, which is comparable with what was observed with control SCNT embryos (Fig. 5d and Supplementary Fig. S7g and h). However, *Kdm4b*-injected SCNT embryos exposed to Wnti treatment showed a great increase in the rosette-like formation rate, from 5.4% to 21.3% (Fig. 5d). We therefore performed RNA-seq and analyzed the expression level of pluripotency genes for the EPI of *Kdm4b*-injected SCNT embryos at the E4.5 stage (Fig. 5e). The GSEA results revealed a pluripotency expression pattern similar to that of the control SCNT embryos (Supplementary Fig. S7i). The PCA analysis results also showed that the *Kdm4b*-injected SCNT samples were grouped together with the control SCNT samples but remained separate from IVF and Wnti-SCNT samples (Supplementary Fig. S7j), indicating a failure to induce the naïve-to-primed transition.

The ectopic expression of *Xist* in pre-implantation embryos is another major barrier affecting post-implantation development of SCNT embryos, and prompted us to investigate the functional relationship between ectopic *Xist* expression and Wnt activation [7,41]. We used CRISPR-Cas9-mediated gene editing to generate *Xist* KO mice (Supplementary Fig. S7k and l), whose deletion region was genotyped similarly to previous reports [7]. Morphology and Wnt reporter activity examination for the EPI of *Xist* heterozygous KO cumulus-derived SCNT embryos at the late-E4.5 stage revealed similar observations to that of the control SCNT embryos (Supplementary Fig. S7m and n). Likewise, transcriptome analysis revealed the expression patterns of pluripotency factors where the transition from the naïve to primed state was also delayed in the EPI of *Xist* KO SCNT embryos, similar to control SCNT embryos. This was further confirmed by GSEA and PCA (Fig. 5f and Supplementary Fig. S7i and j).

Notably, applying a combined approach using *Xist* KO donor cells coupled with *Kdm4d* mRNA injection could significantly improve the development of SCNT embryos. Therefore, through reanalyzing the recently published transcriptome datasets of IVF embryos and SCNT embryos generated by combined approaches (*Kdm4d* injection and *Xist* KO donor) (Fig. 5g) [42], we found that downregulated genes in the SCNT group were associated with the top catalogs of known imprinting regulation and negative regulation of Wnt signaling, indicating disorders of Wnt activity existing even in SCNT embryos treated with combined approaches.

Next, we further examined the development efficiency *in vivo* by manipulating Wnt inhibition coupled with other known correction protocols and developed multiple combined approaches. Given that combining *Kdm4b* mRNA injection with si*Dnmt3a/b* can improve the developmental capacity of SCNT embryos, as we previously reported [12], they were further combined with Wnt inhibition at E3.0. Notably, following blastocyst transfer, the full-term development rate was further increased from 9.1% to 17.5% (Fig. 5c and h, Supplementary Table S1). Moreover, we generated SCNT embryos using *Xist* KO Sertoli cells as donors, and 2.8% of transferred blastocysts reached term (Fig. 5i). However, when these embryos were treated with Wnti at E3.0, the full-term rate was further increased to 12.7% (Fig. 5c and i). Significantly, when we applied a novel combined approach by using *Xist* KO Sertoli donor cells and injecting si*Dnmt3a/3b* and manipulating Wnt inhibition at E3.0, over 20% of SCNT blastocysts that were transferred to the surrogate mothers could reach term (Fig. 5i). The above results indicate that manipulating Wnt inhibition has possible synergistic effects with other reported correction approaches.

### Wnt inhibition improves the lineage development trajectories of SCNT embryos in peri-implantation development

**Figure 6.**
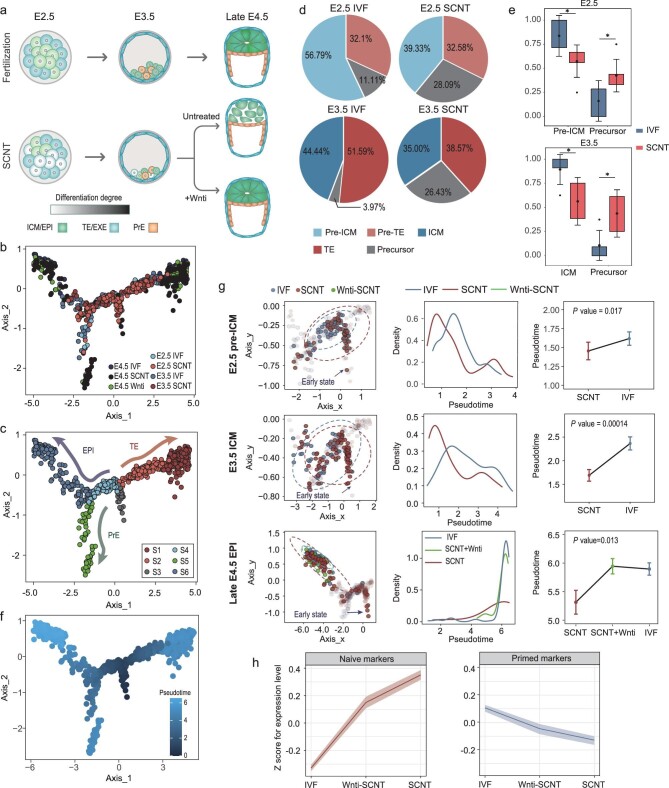
The Wnt inhibitor improves the lineage developmental trajectories of SCNT embryos in peri-implantation development. (a) Schematic representation of the peri-implantation development fates of the IVF and SCNT embryos (Wnti treatment or untreated) with the first two waves of cell fate decisions. The darkness of the color indicates the differentiation degree of cell lineage in embryos. The E2.5 cells, E3.5 ICM cells and late-E4.5 EPI cells from IVF and SCNT embryos were prepared for the scRNA-seq. (b) Projection of the scRNA-seq cells during E2.5, E3.5 and late-E4.5 IVF and SCNT embryos by using the Monocle2 DDRTree method. (c) Trajectory inference for cell lineages during morula-to-late-E4.5 blastocyst transitions of IVF and SCNT embryos by using the Monocle2 DDRTree method. (d) Distribution of IVF and SCNT cells to the different cell lineages during blastocyst formation (E2.5–E3.5) based on trajectory inference. (e) Comparison of the percentage of precursor and ICM cells per IVF and SCNT embryo at E2.5 and E3.5 (*n* = 6 in each group). (f) Visualization of pseudotime value for the cells derived from IVF and SCNT embryos during E2.5 to late E4.5. (g) Comparison of the pseudotime stage of cells derived from IVF-, SCNT- and Wnti-SCNT embryos at E2.5 to late E4.5 along the EPI lineage, showing: the cell distribution and ellipse plot with 95% confidence along the pseudotime axis (left); the distribution of pseudotime values inferred from trajectory analysis (middle); and a comparison of pseudotime stages (right). *P*-values were determined using an unpaired two-tailed *t*-test and means ± SE are shown. (h) Cluster of naïve (*n* = 11) and primed (*n* = 11) genes differentially expressed in IVF-, SCNT- and Wnti-SCNT cells at late E4.5. Shaded regions indicate the standard error of the mean (SE) by calculating the normalized expression for each type of embryo. For (e), *P*-values were determined using the unpaired two-tailed t-test; error bars and means  ± SD.

To detect potential defects occurring in the lineage specification process at the pre-implantation stage, we generated scRNA-seq data of SCNT and IVF embryos at the E2.5, E3.5 and late-E4.5 stages and Wnti-SCNT embryos at the late-E4.5 stage (Fig. 6a), which pinpointed the first two waves of cell fate decisions *in vivo*. We delineated cells’ heterogeneity through dimensionality reduction analysis in line with the development stage, lineage specification and reprogramming strategies (Supplementary Fig. S8a–d).

Further trajectory analyses recapitulated the development paths in six sequential distinct cellular states (S1–S6), which illuminated the trophectoderm (TE)/ICM separation from the precursor state (S3 to S2 and S4) and the bifurcations between EPI and PrE derived from the ICM group (S4 to S5 and S6) (Fig. 6b and c). Assignment of the states to the EPI and ExE branches was supported by the expression of cell-type-specific markers such as *Sox2, Sox17* and *Eomes* (Supplementary Fig. S8e). For TE/ICM segregation (early E2.5 and E3.5), we observed that a greater proportion of cells derived from SCNT embryos remained stuck in the precursor state (S3) and fewer cells entered into the ICM state (S4) than cells in IVF embryos (Fig. 6d). We further measured the cell distribution for each lineage per embryo and obtained the same results (Fig. 6e), suggesting a greatly reduced capacity for lineage specification in SCNT pre-implantation embryos, especially for the ICM lineage.

To track the progression of cells into EPI fates (late E4.5), based on their distribution on the pseudo-time axis (Fig. 6f), we observed a bias of cells in the SCNT embryos within early pseudo-time stages during ICM to EPI differentiation, compared with cells in the IVF and Wnti-SCNT embryos, suggesting that the differentiation delay of cells in SCNT embryos was sustained to late-E4.5 EPI (Fig. 6g). Furthermore, cells of SCNT embryos in EPI fates exhibited higher enrichment in naïve signatures and lower enrichment in primed signatures than IVF cells, whereas Wnti-SCNT cells displayed a more advanced pluripotent state, in agreement with the corrected pluripotency transitions observed in bulk RNA-seq (Fig. 6h and Supplementary Fig. S8f). The results supported that the bias established in the ICM lineages by the pre-implantation stages contributed to the subsequent development of aberrant patterning in SCNT peri-implantation embryos.

## DISCUSSION

SCNT embryos can develop to blastocyst stage with a high efficiency comparable to IVF embryos by overcoming critical epigenetic barriers [5,8–10]. However, most SCNT embryos transferred are arrested at the peri-implantation stage [17,18], indicating the possibility that unidentified barriers prevent the normal implantation of cloned blastocysts. Currently, peri-implantation embryo development is not well understood due to technical limitations. Here, we develop a Matrigel-packed 3D IVC system for achieving the visualization of the peri-implantation development of SCNT embryos *in vitro*. Compared to flattened embryos from 2D culture systems, the 3D IVC system enables us to genuinely evaluate the developmental landscape and lineage specification of embryonic and extra-embryonic structures. Extra-embryonic tissue defects are considered to be an important cause of the abnormal development of nuclear transfer embryos [43]. Our findings show that the rosette-like structure of EPI in SCNT embryos at the peri-implantation stage is extremely disorganized, which highlights the profound impact of defective embryonic tissues on SCNT embryonic development.

Previous studies have mainly focused on correcting the developmental defects of SCNT embryos during the somatic-to-embryonic transition process [5]. However, cell transit in embryos, from a pluripotent state to a fate-committed state, is also a crucial developmental step [20]. However, in normal embryonic development, the naïve pluripotency state is transient before implantation, where key naïve transcription factors such as Klf2, Tfcp2l1, Tbx3 and Esrrb can cross-regulate mutual expression via positive feedback loops to resist exiting the naïve state and counter the establishment of apical-basal polarity [35,44]. Our results, based on different perspectives, reveal that the naïve-to-primed pluripotency of EPI failed to transition in SCNT peri-implantation embryos, regardless of the donor cell type, and the pluripotency transition deficiency further led to defective EPI transformation. Importantly, morphological and molecular deficiencies could still be hard to overcome by correction of soma-persisting H3K9me3 and ectopic *Xist* expression at the pre-implantation stage, indicating that the pluripotency transition deficiency is a common defect of cloned embryos upon implantation.

Dramatic morphological and transcriptional changes are accompanied by extensive epigenetic reprogramming at the time of implantation [31–33]. Following SCNT embryo implantation, the distinct gene expression patterns of EPI between SCNT and IVF may be attributed to another round of epigenetic reprogramming abnormalities. Our ULI-NChIP-seq data exhibit aberrant H3K27me3 deposition on these pluripotency genes. Besides that, it is possible that other epigenetic modifications with abnormal reprogramming have a negative impact on the precise regulation of gene expression.

Finally, we demonstrate that persistent activation of Wnt signaling is an important barrier causing the peri-implantation defects of EPI in the SCNT embryos by interfering with the expression of key pluripotency factors, which should be independent of other known epigenetic barriers. We find that overcoming this barrier by manipulating Wnt signaling inhibition could significantly facilitate the naïve-to-primed pluripotency transitions and their H3K27me3 deposition patterns and further improve peri-implantation development. Importantly, when these Wnti-SCNT blastocysts are allowed to complete full-term development, up to 10.3% of them develop to term. By employing multiple combined protocols, we optimized a novel combined approach, and the overall full-term rate was improved to more than 20%. We tried to generate cloned mice by combining Wnt inhibition with all known strategies (*Kdm4b* overexpression + *Xist* KO donor + si*Dnmt3a/b* + Wnti), but it was hard to get a higher full-term rate. The reason for this may be that the effect of promoting the pre-implantation development of SCNT by *Kdm4b* injection is largely countered by SCNT blastocyst transfer.

**Figure 7.**
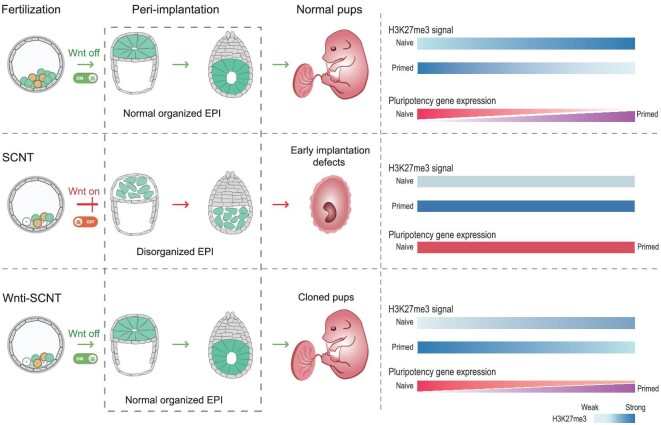
The barrier mechanism and the rescue schemes of the peri-implantation development of cloned embryos: a schematic model showing the defective lineage specification reprogramming in SCNT embryos at the pre-implantation stage. Persistent Wnt activity blocks the naïve-to-primed pluripotency transition of EPI in peri-implantation cloned embryos, accompanied by the aberrant H3K27me3 reprogramming. The barriers can result in EPI disorganization and implantation failure. Wnt manipulation can drive pluripotency transitions of EPI cells by restoring H3K27me3 reprogramming to a considerable degree, further improving the peri-implantation development of cloned embryos.

Wnt signaling is involved in a large variety of important cellular processes such as cell fate decisions, uterine receptivity and cellular metabolism [35,37,45]. In particular, during the process of early embryo development, Wnt signals take part in embryo diapause and pluripotency regulation in embryonic stem cells [34,35]. Previous reports have indicated that canonical Wnt signaling is required in a precise and regulated manner, which might play a dual role in the peri-implantation (E3.5–E5.25) and post-implantation (after E5.5) stages respectively. In different mouse pluripotent stem cell models, Wnt signaling plays different roles: it maintains pluripotency in naïve ES cells, whereas it promotes differentiation in formative and primed ES cells [27,46,47]. When combined with our present findings, we proposed a model that Wnt signaling functions as a molecular switch, the fine-tuning of which is crucial to ensure blastocyst competency for implantation (Fig. 7). However, the persistent activation of the Wnt signaling of EPI causes SCNT blastocysts to remain in the switched-off state, thus blocking pluripotency transitions of EPI and the establishment of epithelial polarity, and contributing to implantation failure. Wnt signals are active before the blastocyst stage [34,48–51]. To evaluate the effect of persistent Wnt signaling, trajectory analyses further demonstrates that the lineage specification process of the SCNT embryos is defective and impedes the developmental progression of SCNT cells, suggesting that the diminished potential of lineage specification maintains Wnt activity of the EPI lineage, whereas the retardation of developmental progression could be resolved by manipulating Wnt signals.

Another important consideration is what factors contribute to the persistent Wnt activation in SCNT embryos. Our results show that numerous genes responsible for the inhibition of Wnt signals, including *Dkk1* [52], *Sox17* [53] and *Tcf7l1* [54], are seriously repressed in SCNT blastocysts, and that the H3K27me3 at the promoter of *Dkk1* was resistant to reprogramming in donor somatic cells after SCNT, suggesting that abnormal epigenetic reprogramming in the donor somatic cells is likely the cause of the dysregulation of these Wnt antagonistic genes in SCNT embryos. Subsequent epigenetic regulation of Wnt antagonistic genes needs to be further investigated.

Wnt signaling is evolutionarily conserved from metazoans to vertebrates [55]. The regulation of functional canonical Wnt signaling has also been observed upon implantation of bovine, porcine, primate and human blastocysts [34,56–60]. Specifically, recent studies reported that supplementation of DKK1 (a secretory inhibitor of canonical WNT signaling) in the embryo culture media can increase blastocyst formation and the live birth rate of cloned buffalo embryos [61], indicating that the molecular mechanism of underlying persistent Wnt activation in SCNT-derived embryos is likely conserved among mammals. Thus, unlike approaches that require additional genetic editing or micro-injection procedures, our study provides a simpler and more efficient approach for enhancing commercial animal breeding and regenerative medicine.

## CONCLUSION

Here we recapitulated the peri-implantation embryonic development of SCNT embryos by developing a 3D *in vitro* culture system. Our findings reveal that the EPI transformation events are defective, which is caused by the diminished lineage specification capacity of SCNT blastocysts, which is linked with blocked naïve-to-primed pluripotency transitions and aberrant H3K27me3 remodeling. With the advantage of the Wnt reporter system, we further identified that persistent Wnt activity as a critical peri-implantation barrier impedes pluripotency progression and morphogenesis of EPI. Overcoming this barrier by manipulating Wnt signals could not only fix these defects and promote pluripotency progression, but also greatly improve cloning efficiency.

## MATERIALS AND METHODS

### *In vitro* 3D culture of mouse embryos

IVC mediums used from Ma. *et al.*’s optimized 2D culture system could significantly increase the egg cylinder formation rate. E3.5 mouse blastocysts were digested with 0.5% Pronase E (Sigma, P8811) to remove the zona pellucida, and transferred into the culture dishes. Each blastocyst was cultured in a 50 μl IVC1 medium droplet in a 35 mm petri dish covered with mineral oil. To modify a 3D condition for embryo development, 25% Matrigel was injected into prewarmed IVC1 droplets by using a glass capillary, which resulted in cultured mouse embryos becoming embedded. In under a minute, the Matrigel would solidify and suspend embryos in the medium. Then the culture dishes were placed at 37°C under 5% CO_2_ in air for 2 days. After 2 days of culture, embryos developed to E5.0–E5.25 and formed 3D rosette or early lumen structures. Then the IVC1 medium was removed with a pipette and replaced with equilibrated IVC2 medium for 1–2 days of culture. At approximately day 3–4 of *in vitro* culture, early egg cylinders should emerge (E5.5). The *in vitro* cultured E5.5 mouse embryos were similar in structure and size to *in vivo* E5.5 mouse embryos. The following is the components of IVC mediums: CMRL 1066 basic medium (Gibco, 11530037) supplemented with 10% FBS (Millipore, ES-009B), 1 mM sodium pyruvate (Invitrogen, 11360070), 1 : 100 diluted N2 supplement (Invitrogen, 17502048), 1 : 200 diluted B27 (Invitrogen, 17504044) and 100 units ml-1 penicillin-streptomycin (Invitrogen, 15140122). IVC2: 20% FBS, 1 mM sodium pyruvate, 1 : 100 diluted N2 supplement and 1 : 200 diluted B27, 100 units ml-1 penicillin-streptomycin.

### SCNT and Wnti treatment

Recipient MII oocytes were used from 8–10-week-old B6D2F1 female mice. At 14 hours after the hCG injection, MII oocytes were enucleated in HCZB medium containing 5 mg/mL cytochalasin B (CB) (Sigma, C6762) by using a Piezo-driven micromanipulator. The wild-type cumulus, Sertoli, ES cells or Xist KO cumulus or Sertoli cells were used as nuclear donors, and transferred into enucleated oocytes by using a Piezo-driven micromanipulator. Then the reconstructed SCNT oocytes were incubated in CZB medium for 1 hour. For activation treatment, the reconstructed SCNT oocytes were activated by 5 hours incubation in 1 mM SrCl2 in Ca^2+^-free CZB and 5 μg/mL cytochalasin B. Activated embryos were washed and cultured in G-1 Plus medium at 37°C under 5% CO_2_ in air. Small-molecule inhibitors IWP2 (Selleck, 686770-61-6), IWR1-endo (Selleck, 1127442-82-3) and Wnt activator CHIR99021 (Sigma, SML1046) were applied to inhibit or activate the canonical Wnt/β-catenin signaling pathway. To inhibit the Wnt signaling pathway, SCNT embryos were respectively transferred to G-1 PLUS containing Wnt inhibitor IWP2 (2 μmol/ml) or IWR1-endo (10 μmol/ml) from G-1 PLUS medium at E1.5–E3.0, E1.5–E4.5 or E3.0–E4.5. For *in vivo* embryo transfer, SCNT embryos or IVF embryos were treated with IWP2, IWR1-endo or IWP2 + IWR1-endo at E3.0 (late morula)–E4.0 (blastocyst). To activate the Wnt signaling pathway, SCNT or IVF embryos were cultured in G-1 PLUS containing Wnt activator CHIR99021 (3 μmol/ml).

## Supplementary Material

nwad173_Supplemental_Files

## Data Availability

Further information and requests for resources and reagents should be directed to, and will be fulfilled by, the lead contact, Shaorong Gao (gaoshaorong@tongji.edu.cn). This study did not generate new unique reagents. ULI-NChIP-Seq data, scRNA-seq data and Smartseq2 RNA-seq data generated in this study have been deposited in the Genome Sequence Archive (GSA) (https://bigd.big.ac.cn/gsa/) of the China National Center for Bioinformation-National Genomics Data Center (CNCB-NGDC) under accession code: CRA006661. This paper does not report original code. Any additional information required to reanalyze the data reported in this paper is available from the lead contact upon request.
