# Supplementary material for: Inhibition of Wnt activity improves peri-implantation development of somatic cell nuclear transfer embryos
Source: Natl Sci Rev. 2023 Aug 16;10(9):nwad173. doi: 10.1093/nsr/nwad173 (PMC10430793; doi:10.1093/nsr/nwad173)
Supplement: nwad173_Supplemental_Files [file nwad173_supplemental_files.zip › NSR supplementary data .docx]

**SUPPLEMENTARY DATA**

**SUPPLEMENTARY MATERIALS AND METHODS**

**Animals**

The specific pathogen-free mice were housed in the animal facility of Tongji University, Shanghai, China. The use and care of animals complied with the guideline of the Tongji University Guide for the use of Laboratory Animals. Female B6D2F1 mice (8-10 weeks old) were used for embryos collection. Male B6D2F1 mice (10-12 weeks old) were used for sperm extraction. Female ICR mice (8-10 weeks old) were used for embryo transplantation.

**Embryo collection**

MII oocytes were obtained from 8-week-old B6D2F1 females super-ovulated by injection with 5 IU each of pregnant mare serum gonadotropin (PMSG), followed by injection of 6 IU of human chorionic gonadotropin (hCG) (San-Sheng Pharma-ceutical) 48 hours later. Naturally fertilized zygotes were obtained from the ampulla of the uterine tube of mice. The embryos were cultured in CZB medium at 37°C under 5% CO2 in air for 2 hours.

**In vitro fertilization**

At 14 hours after hCG injection, the cumulus-oocyte complexes were released from the oviducts of female B6D2F1 mice. MII oocytes were obtained from the ampulla of the uterine tube. Sperm collected from the cauda epididymis of adult B6D2F1 male mice or DBA2 male mice was incubated in G-IVF medium (Vitrolife) for 20-30 min to allow sperm to swim out. Activated sperm and oocytes were placed in the G-IVF droplets for 3-4 hours, then these embryos were washed and cultured in G-1 PLUS medium (Vitrolife).

**In vivo uterus transplantation and cesarean section**

E3.5 or E4.0 control or Wnti treated SCNT and IVF embryos were transferred into uterine horns of pseudopregnant ICR female mice at 2.5 d.p.c or 3.5 d.p.c. Cesarean section was performed at E7.5 or E19.5. The pups and placenta were weighed and photographed. The surviving pups were nursed by lactating ICR females.

**In vitro transcription of Kdm4b mRNA and microinjection**

T7 promoter was cloned into Kdm4b, and mRNAs were synthesized in vitro using mMESSAGE T7 ULTRA kit (Life Technology) following the manufacturer’s instructions. The microinjection concentration of Kdm4b mRNA was 100 ng/µl. Enucleated oocytes were injected with ~10 pL of mRNA using a Piezo-driven micromanipulator.

**Knockdown of Dnmt3a/b in SCNT embryos**

siRNAs against Dnmt3a and Dnmt3b were synthesized as described previously [1]. siRNA was diluted in nuclease free water at a final concentration of 10 µM stock solutions. Enucleated oocytes were injected with ~10 pL of 10 µM siRNAs for targeting Dnmt3a and Dnmt3b using a Piezo-driven micromanipulator. After incubating for 30 min in CZB medium, the nuclei of donor cells were transferred into the pre-treated enucleated oocytes by direct injection.

**Generation of the TCF/LEF-H2B-H2B-GFP reporter mouse embryos**

TCF/LEF-H2B-GFP reporter mouse embryos were generated with the piggyBac transposon system, PBase mRNAs were synthesized in vitro using mMESSAGE T7 ULTRA (Life Technology). The mixture of PBase mRNA (150 ng/µl) and TCF/LEF-H2B-GFP reporter donor plasmid (20 ng/µl) was injected into the cytoplasm of MII oocytes at a dose of ~10 pL/times by using a Piezo-driven micromanipulator. The knock-in efficiency of TCF/LEF-H2B-GFP elements is nearly 100%.

**Real time RT-PCR analysis**

To analyze the expression of naïve and primed genes in E4.5 SCNT and IVF embryos, total RNA of several embryos were isolated by TRIzol reagent. The cDNA was reverse transcripted using 5 X All-in-One RT MasterMix (abm). Quantitative RT-PCR was carried out on a ABI7500 Real-Time PCR System (Applied BioSystems) using a SYBR Premix Ex Taq (Takara) mix. Relative quantification was done using Gapdh as a reference gene (Table. S2).

**Blastocyst complementation assay**

8-10 ICM cells from IVF and SCNT blastocysts were respectively injected into SCNT blastocysts by using a Piezo-driven micromanipulator. Around 10-20 injected embryos were transferred into both uterine horns of pseudopregnant ICR female mice at 2.5 d.p.c. Caesarian section was performed at E7.5. The embryos obtained were photographed and fixed in 4% paraformaldehyde (PFA) for immunostaining.

**Immunostaining**

Embryos were fixed with 4% PFA for 30 min at room temperature (RT) and washed twice in 0.5% Bovine Serum Albumin (BSA, Thermo Fisher Scientific) in PBS. The fixed embryos were permeabilized with 0.5% Triton-X-100 for 30 min and then washed twice. Then, the samples were blocked with 3% BSA in PBS for 1 hour at RT, and incubated with primary antibodies in block solution overnight at 4°C. After washing three times, samples were incubated with secondary antibodies for 2 hours at RT, and nuclei were incubated with DAPI (Sigma, 62248). Embryos were then washed three times. Embryos were observed under a confocal microscope LSM 880 (Carl Zeiss).

**Isolations of Lineage-specific cell sample at peri-implantation stage**

As the embryo develops to late E4.5, four major lineages (Epi, PrE, Exe and TGC) have emerged. There are apparent differences between their cell morphology and cell junctions. In order to obtain Epiblast cells, these embryos were first digested in tryptic the enzyme (TE) until the cells were loose. Then, the Epi and PrE lineage were separated from TGC and Exe lineage by gentle pipetting using a fire-polished glass needle with an inner diameter of 200 μm after transferring into the HCZB medium. Finally isolate the individual Epi cells and a gobbet of PrE cells was isolated by gentle pipetting using a 30 μm glass microneedle with a Piezo-driven micromanipulator. Individual Exe cells and a gobbet of TGC cells were obtained by using the same methods. The isolated cells in four lineages were sampled for Smart-seq2 (Supplementary information, Fig. S3a, b). Among them, the classical markers for epiblast (EPI), extraembryonic ectoderm (EXE/TGC) and primitive endoderm (PrE) were used to evaluate the quality of the isolated manipulation. By cluster analysis based on the gene expression, lineage identities and associated the lineage-specific markers could be rigorously interrogated.

**Sample harvest for Smart-seq2, Single Cell RNA-seq, ChIP-seq and WGBS**

For cleavage-stage embryos, the zona pellucidae of the embryos were digested with 0.5% pronase E and the embryos were then transferred into 0.5% BSA in PBS solution. Polar bodies were removed by gentle pipetting using a glass capillary. For ICM and trophectoderm isolation, the zona pellucidae of blastocysts were digested with 0.5% pronase E. The blastocysts were incubated in Ca^2+^-free CZB for 20 min, and then gently pipetted by using a pipette to separate the trophectoderm cells and ICM cells. For EPI and EXE isolation of peri-implantation embryos, embryos were first pipetted by using a glass capillary to separate from matrigel in HEPES-buffered CZB medium, and then digested in tryptic enzyme (TE) for 10 min. Single cells were separated by using a glass capillary with an inner diameter of 20 µl.

The Smart-seq2 method followed previously published studies [2]. Briefly, about 10 cells were used per reaction, and two or three replicates were performed for each group. All isolated cells were washed three times in 0.5% BSA in PBS solution, and transferred into a tube containing lysis buffer. Each tube was mixed thoroughly and centrifuged briefly and incubated at a temperature of 70°C for 3 min. Reverse transcription was performed directly on the cytoplasmic lysate of indicated samples. The total cDNA library was then amplified by 19 cycles for the library construction. The sequence libraries were generated using the KAPA Hyper Prep Kit according to the manufacturer’s instructions. Paired-end 125-bp or 150-bp sequencing was further performed on a NovaSeq (Illumina) at Berry Genomics Corporation.

For ULI-NChIP-seq, 200-500 cells were used per reaction, and two or three replicates were performed for each sample. All isolated cells were washed three times in 0.5% BSA in PBS solution to avoid possible contamination. The ULI-NChIP procedure was performed as previously described [3-5]. 1µg histone H3K27me3 antibody was used for each immunoprecipitation reaction. The sequence libraries were generated using the KAPA Hyper Prep Kit according to the manufacturer’s instructions. Paired-end 125-bp or 150-bp sequencing was further performed on a NovaSeq (Illumina) at Berry Genomics Corporation.

For WGBS, about 100 cells were used per reaction. All isolated cells were washed three times in 0.5% BSA-PBS solution to avoid possible contamination. The sequencing libraries were generated using the Pico Methyl-Seq Library Prep Kit following the manufacturer’s instructions. Paired-end 125-bp or 150-bp sequencing was further performed on a NovaSeq (Illumina) at Berry Genomics Corporation.

**Single cell RNA-seq library preparation and sequencing**

Single cell RNA-seq (scRNA-seq) library construction was preformed following our previously reported protocol [6, 7]. Briefly, using mouth pipetting, single cells were placed in a 200μl tube containing lysis buffer and lysed at 72°C for 3 min. The reverse transcription mixture was added to each tube and incubated at 25°C for 5 min, followed by 42°C for 60 min, 50°C for 30 min, and 70°C for 10 min in a thermocycler. Then we performed the cDNA amplification and purification by AMPure XP beads. Subsequently, Biotin PCR was carried out to enrich the available DNA fragments. Finally, according to the KAPA Hyper Prep Kits manual, the single cell RNA-seq library was prepared and sequenced with 150bp pair-end reads on Illumina Hiseq.

**RNA-seq data analysis**

The raw data of public RNA-seq data for naïve, rosette and primed mouse embryonic stem cells were download from NCBI (GEO GSE105762). Next, the in-house and public RNA-seq reads were quality filtered by trim_galore (version 0.6.4_dev, https://www.bioinformatics.babraham.ac.uk/projects/trim_galore/) then aligned to mouse reference (mm10) using the alignment tool STAR (version 2.7.3a, https://github.com/alexdobin/STAR) with parameter “–outFilterMultimapNmax = 1”. Expression levels for genes were quantified by using featureCounts (version 2.0.0, http://subread.sourceforge.net/) via counting the reads located on the exons annotated by gene annotation file (GTF) from Ensemble (Mus_musculus.GRCm38.93.gtf) and normalized with transcripts per million (TPM). Principal component analysis was performed using ‘prcomp’ function, and hierarchical cluster analysis was conducted with ‘hclust’ function in R (version 4.0.3) with TPM. Differentially expressed genes (DEGs) were identified using the DESeq2 (version 1.30.1, <https://bioconductor.org/packages/release/bioc/html/DESeq2.html>), Benjamini-Hochberg adjusted p-value < 0.01 and absolute log2FoldChange >1 were used to screen the significant DEGs, furthermore low expressed genes which average TPMs were less than 0.5 in both case and control group were excluded. The public data represented naïve, and primed state in vivo were download from NCBI (GEO GSE100597) and the count matrix was used directly. The DEGs of from all pairwise comparisons with aforementioned cut-offs. The average TPM values of all DEGs for each group was calculated, then classified into 15 clusters by k-means algorithm (Table. S3). We selected representative clusters for transcriptome alterations between epiblasts of IVF and SCNT embryos at late E4.5 stage. Cluster 1 and 4 were referred to ‘E4.5 EPI of IVF embryos specific downregulated or upregulated genes’, cluster 2 and 3 were ‘E4.5 EPI of SCNT embryos specific downregulated or upregulated genes’. Functional enrichment analysis for these genes were conducted on the Metascape (<https://metascape.org/>) with GO biological processes database.

**Gene Set Enrichment Analysis (GSEA)**

First, the DEGs between naïve and primed mouse embryonic stem cells from public data (GEO GSE105762) with Benjamini-Hochberg adjusted p-value < 0.01 and absolute log2FoldChange >1. The top 200 up regulated DEGs for naïve and primed mESCs were selected and represented naïve and primed state (Table. S4). Using the generated gene sets as input gene matrix transposed file, we performed gene set enrichment analysis (GSEA, version 4.1.0, <https://www.gsea-msigdb.org/>) for all kinds of SCNT embryos by comparing with IVF ones by using default parameters.

**Single cell RNA-sequencing (scRNA-seq) analysis**

scRNA-seq data were processed following single-cell tagged reverse transcription sequencing (STRT-seq) protocol, and the barcode information for each cell was listed in Table. S5. Briefly, read 2 of the fastq files contained 8 bp cell barcodes and 8 bp unique molecular identifiers (UMIs), the barcodes and UMIs were split and switched to the identifier line of paired read 1. Next the read 1 were trimmed to remove the template switch oligo (TSO) primer, low quality bases and polyA sequence. Trimmed reads were aligned and expression levels for genes were quantified with same reference, gene annotation file, alignment tool and quantification tool used in bulk RNA-seq. The expression levels for scRNA-seq were normalized with counts per million (CPM) due to 3′ end bias characters of scRNA-seq. The generated counts matrix was further filtered based on the following criteria: 1). sex chromosome genes were excluded to eliminate gender effect due to sequenced cells were derived from embryos with distinct genders; 2). cells which detected less than 3000 genes and genes which detected by less than 10 cells were excluded; 3). the ratio of UMIs on mitochondrial genes should be below 20% to pass the low-quality cells; 4). cell-to-cell Pearson’s correlations should be more than 0.6 to exclude outlier cells.

**Pseudotime Analysis**

First, we imported the filtered count matrix into Seurat (version 4.0.2, <https://satijalab.org/seurat/>). The data were normalized by sctransform algorithm after removing the variation induced by cell cycle phase scores, mitochondrial mapping percentage, UMIs numbers and gene numbers. Recalibrated data was then used for principal components analysis (PCA) using RunPCA on the top 3 000 highly variable genes with default parameters. Dimensionality reduction was applied to the first ten principal components, and the clusters were identified by construction of a shared nearest neighbor (SNN) graph. Finally, we visualized cell clusters after projecting them into two-dimensional latent space via the uniform manifold approximation and projection (UMAP) algorithm.

We then detected the differentially expressed genes (DEGs) by comparison with the cells derived from IVF embryos at E2.5, E3.5 and late E4.5 stage, for late E4.5 stage, the cells are grouped into Epi, PrE and Exe lineage based on the cluster information aforementioned with representative marker labelling. DEGs were identified by using the FindMarkers function in the Seurat package based on the Wilcoxon rank-sum test, and genes detected in at least 80% of the two compared cell populations were considered. The DEGs with absolute average log transformed fold change >2 and adjusted P value < 0.01 were retained for the candidate ordering genes. Combined with representative marker for mouse embryonic lineage segregation, finally we got a 541 gene list which are critical for the differentiation process (Data S4), especially for embryonic lineages, and these genes were used as ordering genes to construct the development trajectory.

Due to the scRNA-seq data were generated by different batches, batch effect was corrected with the integration module in Seurat. Next, a newCellDataSet object in Monocle (version 2.18.0, https://github.com/cole-trapnell-lab/monocle-release) with raw count matrix was generated by setting expressionFamily parameter to ‘negbinomial.size()’. Then the estimateDispersions function was used to calculate empirical dispersion of each gene in the negative binomial model, the genes were ranked by empirical dispersion value divided by the fitted theoretical dispersion value. In practice, a new Monocle object was created by using the integrated and scaled data by Seurat, the expression Family parameter set as ‘gaussianff()’, then 541 ordering genes based on dispersion estimation as previously described were ranked, from the top 20 to the top 541 of the ordering genes were selected to construct the more than 500 trajectories. Among them, the classical markers for inner cell mass (ICM), trophectoderm (TE), epiblast (EPI) and primitive endoderm (PrE) were used to evaluate the quality of the trajectories. Finally, the top 88 ordering genes were chosen to construct the trajectory by for downstream cell fate analysis.

**WGBS and H3K27me3 ChIP-seq data analysis**

WGBS reads were first processed using trim_galore to trim adaptor and low-quality bases then aligned to the mouse genome build mm10 using bitmapperBS (version 1.0.2.3, <https://github.com/chhylp123/BitMapperBS>), and PCR duplication were removed. The DNA methylation level of each CpG site was calculated using MethylDackel (version 0.3.0, <https://github.com/dpryan79/MethylDackel>). For the CpG sites methylation levels of E3.5_ICM, E4.5_ICM, E5.5_EPI and E6.5_EPI, bedgraph files were download from GEO database at accession number GSE108711 and GSE76505. The CpG locus which covered at by 2 reads and detected by all samples was included for downstream analysis. Top 50% high variable CpG sites were used to PCA analysis. The differentially methylated regions (DMRs) were identified by DSS package (version 2.38.0, <http://bioconductor.org/packages/DSS>) with following parameters: ‘nCG’ >5, ‘diff.Methy’>0.1 ‘areaStat’ >20.

For H3K27me3 ChIP-seq analysis, raw reads were trimmed and aligned to mm10 using the bowtie2(version 2.3.5.1, <http://bowtie-bio.sourceforge.net/bowtie2/index.shtml>). H3K27me3 ChIP-seq raw reads of E3.5 ICM to E6.5 EPI were download from the GEO database at accession number GSE76687 and processed consistently. Unique mapped reads were retained for downstream analysis. The read depth H3K27me3 modification were binned into 1000-bp bins and normalized with RPKM, top 50% highly variable bins were used to process principal components analysis and hierarchical clustering. To compare the genic H3K27me3 coverage for genes, the read depth of promoter and genebody region of each Ensemble gene was calculated and normalized with RPKM by ‘bamCompare’ command in deepTools (version 3.5.1, https://deeptools.readthedocs.io/). The genes with differential deposition of H3K27me3 were identified by t.test and multiple testing using the Benjamini-Hochberg (BH) procedure to control the false discovery rate, the genes which at least covered by 10 reads, and p-value < 0.05, FDR<0.2 were considered as differential H3K27me3-marked genes. Furthermore, the genes which RPKM value in SCNT_E4.5_WNTi fell between the IVF_E4.5 and SCNT_E4.5 samples, and were significantly distinct from SCNT_E4.5’s were termed WNTi repaired H3K27me3-marked genes, which reflected the improvement of SCNT embryos by Wnt inhibition. The signal tracks for each sample were generated by deepTools (version 3.5.1) and visualized by IGV (version 2.12.2, <https://igv.org/>).

**Quantification and statistical analysis**

Statistical analyses were performed by Prism software (GraphPad Software 9) and R software version 4.0.3. Significance is defined as a p-value < 0.05 indicated with asterisk (*p-value <0.05, **p-value <0.01, ***p-value <0.001). The statistical details of the data described in this paper, including the statistical tests used, the value and definition of *n*, and dispersion and precision measures, can be found in the figure legends.

**SUPPLEMENTARY FIGURES**


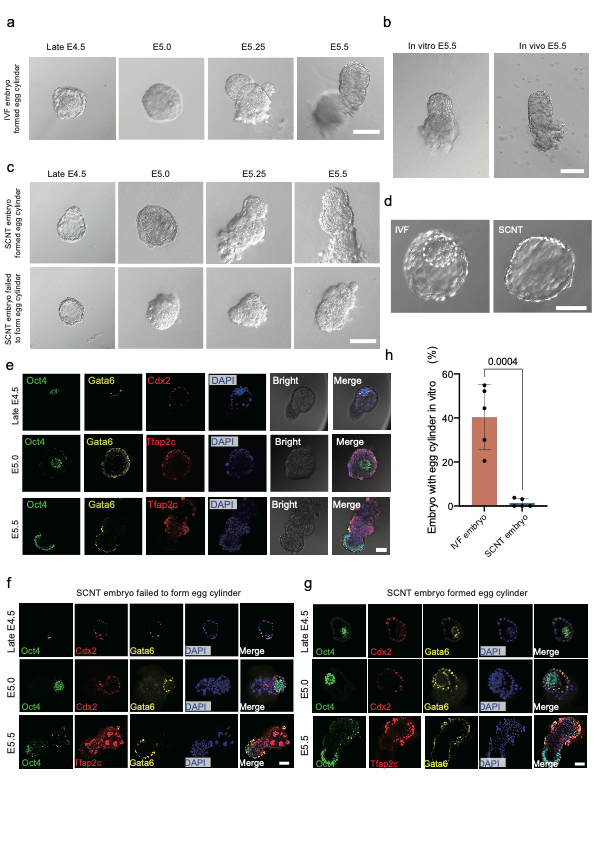


**Supplementary Figure 1. In vitro peri-implantation development defects of SCNT embryos.**

(a) Morphology of the in vitro development of individual IVF embryos. Scale bar, 100 µm.

(b) Comparison of morphology between in vivo and in vitro IVF embryos at E5.5. Scale bar, 100 µm.

(c) Morphology of the in vitro development of individual SCNT embryos. Top: SCNT embryo that formed egg cylinder. Bottom: the SCNT embryo with abnormal peri-implantation development. Scale bar, 100 µm.

(d) Comparison of morphology between IVF and SCNT embryos at late E4.5. Scale bar, 50 µm.

(e) Confocal images of development of IVF embryos at the peri-implantation stage, co-stained for *Oct4*, *Gata6*, *DAPI* and *Cdx2* or *Tfap2c*. Scale bar, 50µm.

(f, g) Confocal images of the aberrant (f) and normal (g) development of SCNT embryos at the peri-implantation stage, co-stained for *Oct4*, *Gata6*, *DAPI* and *Cdx2* or *Tfap2c*. Scale bar, 50µm.

(h) Percentage of the egg cylinder formation in in-vitro cultured IVF and SCNT embryos at E5.5. *p*-values were determined using unpaired two-tailed t-tests; error bars and means ± SD are shown for *n* ≥ 3 biological replicates.


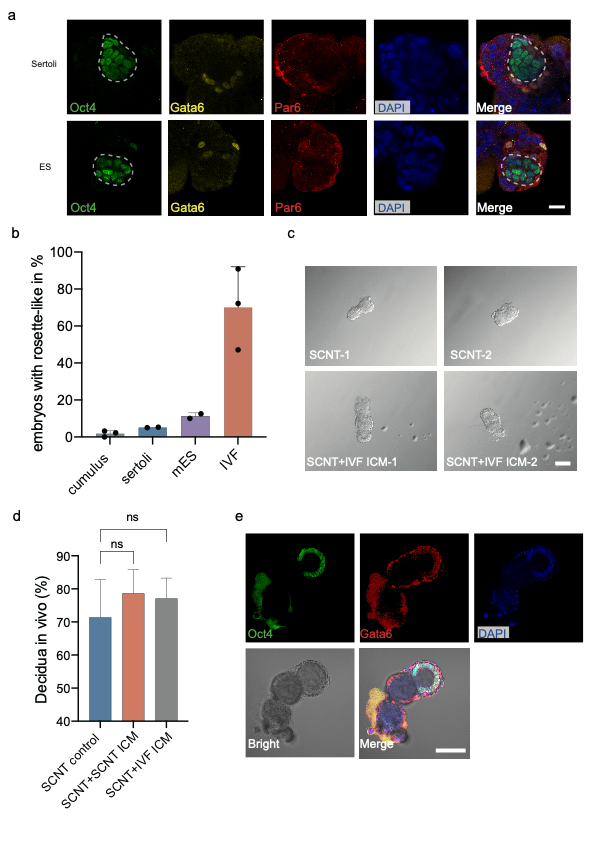


**Supplementary Figure 2. Improving egg cylinder development of SCNT blastocysts complemented with IVF ICM cells.**

(a) Confocal images of the EPI structures in cloned embryos derived from Sertoli and ES cells at E5.0. Embryos were co-stained for *Oct4*, *Gata6*, *Par6* and DAPI. The EPI cells were encircled by a dotted white line. Scale bar, 20 µm.

(b) Percentage of the rosette-like structures formation in the in-vitro cultured IVF and SCNT embryos derived from three types of cells at E5.0. Error bars and means ± SD are shown for *n* = 2-3 biological replicates.

(c) Comparison of morphology at E5.5 between in vitro SCNT embryos complemented with or without IVF ICM cells. Scale bar, 100 µm.

(d) The ratios of decidua (E7.5) in SCNT embryos complemented with IVF and SCNT ICM cells. *p*-values were determined using unpaired two-tailed *t*-tests; error bars and means ± SD are shown for *n* = 3 biological replicates.

(e) Confocal images of structures in E5.5 SCNT embryos complemented with IVF cells. Embryos were co-stained for *Oct4*, *Gata6* and DAPI. Scale bar, 100 µm


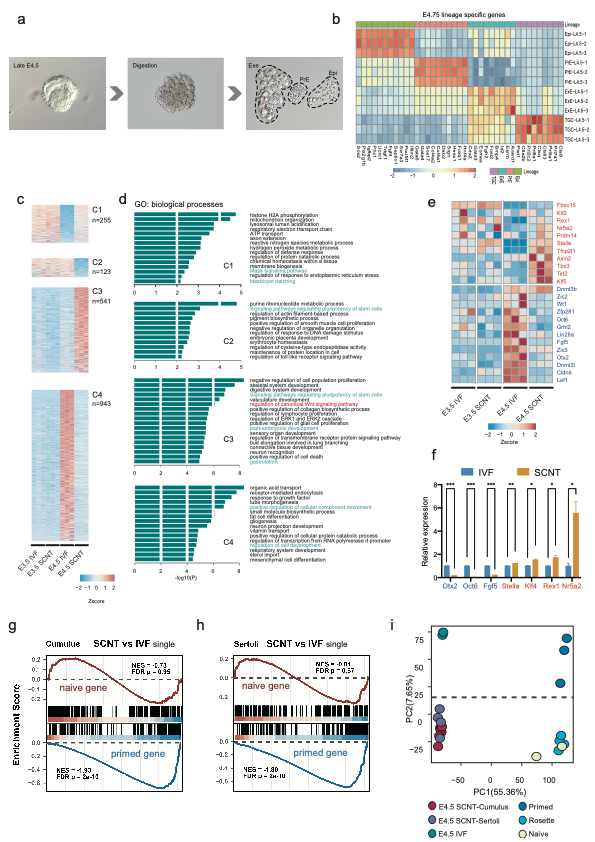


**Supplementary Figure 3. Abnormal naïve to primed transitions of EPI in SCNT embryos.**

1. Isolation process of lineage-specific cells in a late E4.5 embryo.

(b) Heatmap showing the expression of lineage-specific genes in four lineage tissues.

(c) Differentially expressed genes (DEGs) (FC>1.0, adjust *p* value<0.01) between the E3.5 ICM and the late E4.5 EPI of IVF and SCNT embryos.

(d) The enrichment of Gene Ontology (GO) terms of the four classifieds in (A). The categories highlighted were related to embryo development. The *p* values were calculated based on hypergeometric test using Metascape.

(e) Comparison of representative pluripotency markers among the E3.5 ICM and the late E4.5 EPI of IVF and SCNT embryos.

(f) qRT-PCR analysis on differential expression of pluripotency markers between the late E4.5 EPI of IVF and SCNT embryos. *p*-values were determined using unpaired two-tailed t-test; error bars and means ± SD are shown for *n* = 3 biological replicates.

(g, h) GSEA of specific genes expressed in naïve and primed ESCs in the late E4.5 EPI of single IVF and SCNT embryos derived from Cumulus (E) and Sertoli cells (F). NES, normalized enrichment score.

(i) PCA comparison of gene expression profiles among the late E4.5 EPI of IVF, Cumulus and Sertoli cell-derived SCNT embryos, naïve ESCs, primed ESCs and RSCs. Published transcriptomes data of stem cell lines were obtained from GSE145727.

**
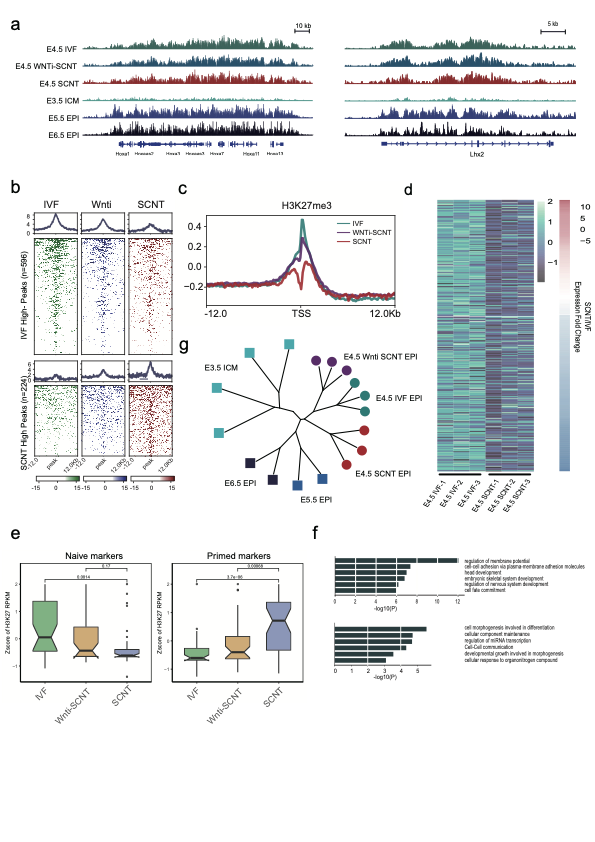
**

**Supplementary Figure 4. Distinct H3K27me3 distribution patterns of EPI in peri-implantation SCNT and IVF embryos.**

(a) The IGV view showing the H3K27me3 signals at developmental genes *Hox* cluster, *Lhx2* in E3.5-E6.5 epiblast. E3.5, E5.5 and E6.5 epiblast data are adopted from previously published datasets (GSE76687).

(b) Heatmap centred over promoters of genes with differential deposition of H3K27me3 between the late E4.5 EPI of IVF, SCNT and Wnti-SCNT embryos.

(c) Comparisons of the global H3K27me3 level centred over promoters of genes between the late EPI of IVF, SCNT and Wnti-SCNT embryos.

(d) Heat maps showing the H3K27me3 levels around promoter and gene body (left) and fold changes of gene expression (right) between the late E4.5 EPI of IVF and SCNT embryos.

(e) The boxplot showing the differential H3K27me3 enrichment in naïve and primed markers between the late E4.5 EPI of IVF and SCNT embryos.

(f) The enrichment of Gene Ontology (GO) analysis of H3K27me3 rescued regions in the late E4.5 EPI of Wnti-SCNT embryos. SCNT high-H3K27me3 rescue-enriched (up), IVF high-H3K27me3 rescue-enriched (down). The *p* values were calculated based on hypergeometric test using Metascape.

**
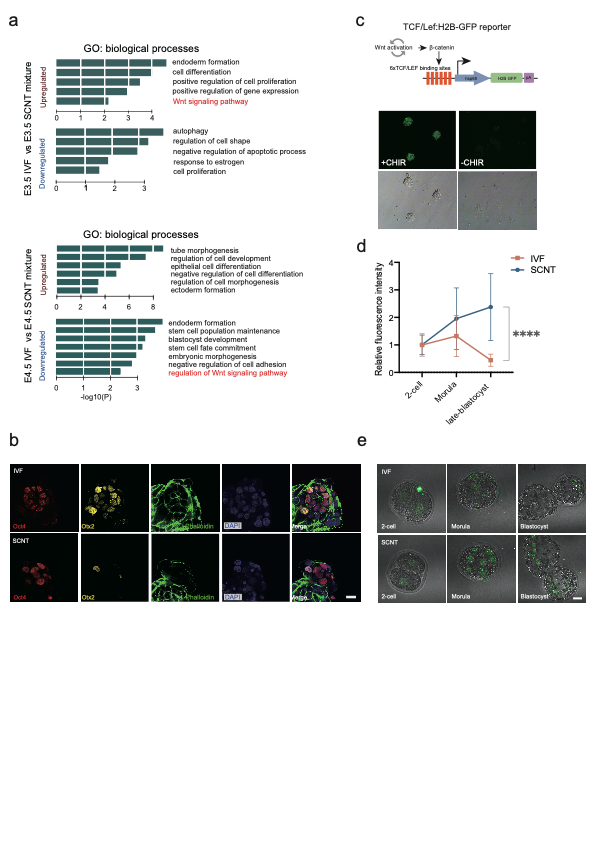
**

**Supplementary Figure 5. Persistent active Wnt signaling in SCNT embryos.**

(a) The enrichment of GO terms of up- and downregulated genes in the E3.5 and late E4.5 EPI of IVF and SCNT embryos. The *p* values were calculated based on hypergeometric test using Metascape.

(b) Confocal images for detecting *Otx2* expression of late E4.5 IVF and SCNT embryos. Embryos were co-stained for *Oct4, Otx2, Par6* and DAPI. Scale bar. 20 µm.

(c) Endogenous Wnt signaling in R1 ESCs detected by TCF/LEF-H2B-GFP reporter with Wnt activator CHIR and no treatment.

(d) Comparison of endogenous Wnt signaling in the pre-implantation IVF and SCNT embryos. *p*-values were determined using unpaired two-tailed *t*-test; error bars and means ± SD are shown for *n* = 3 biological replicates.

(e) Representative images of endogenous Wnt lignals in the preimplantation IVF and SCNT embryos.


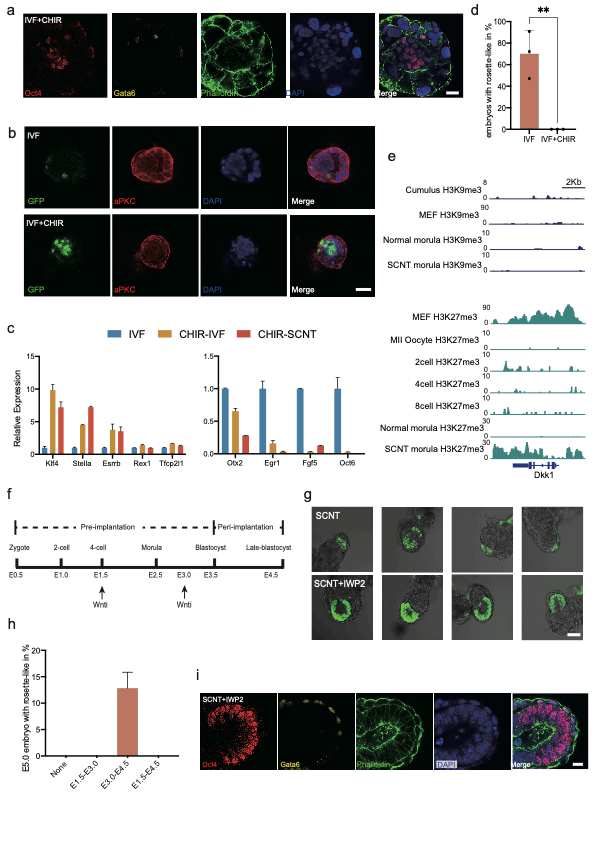


**Supplementary Figure 6. Wnt manipulations for culture of IVF and SCNT peri-implantation embryos.**

(a) Confocal images of the EPI structures in the in-vitro cultured IVF embryos with CHIR and no treatment at E5.0. Embryos were co-stained for *Oct4*, *Gata6*, *Par6* and DAPI. The epiblast was encircled by a dotted white line. Scale bar, 20 µm.

(b) TCF/Lef: H2B-GFP IVF and CHIR treated IVF embryos at E4.5 stained for aPKC and DAPI. Scale bar, 50µm.

(c) QPCR analysis on differential expression of pluripotency markers between the E5.0 EPI of IVF, CHIR-treated IVF, CHIR-treated SCNT embryos.

(d) Percentage of rosette structures formation in the in-vitro cultured IVF embryos with CHIR and no treatment at E5.0. *p*-values were determined using unpaired two-tailed *t*-test; error bars and means ± SD are shown for *n* = 3 biological replicates.

(e) The IGV view showing the H3K27me3 and H3K9me3 signals at *Dkk1* loci in donor somatic cells and early embryo data are adopted from previously published datasets (GSE53939; GSE36292; GSE73952).

(f) Schematic representation of strategies on inhibition of Wnt signals at the pre-implantation stage.

(g) Confocal images of structures in the E5.25 SCNT embryos treated with Wnti at E3.0-E4.5, relative to untreated SCNT embryos. Embryos were stained for *Oct4*. Scale bar, 50 µm.

(h) Percentage of rosette structures formation in the Wnt inhibitor (Wnti) treated embryos at E1.5-E3.0, E3.0-E4.5 and E1.5-E4.5. Error bars and means ± SD are shown for *n* = 3 biological replicates.

(i) Confocal images of the EPI structures in E5.25 SCNT embryos treated with Wnti. Embryos were co-stained for *Oct4*, *Gata6*, *Par6* and DAPI. The EPI cells were encircled by a dotted white line. Scale bar, 20 µm.


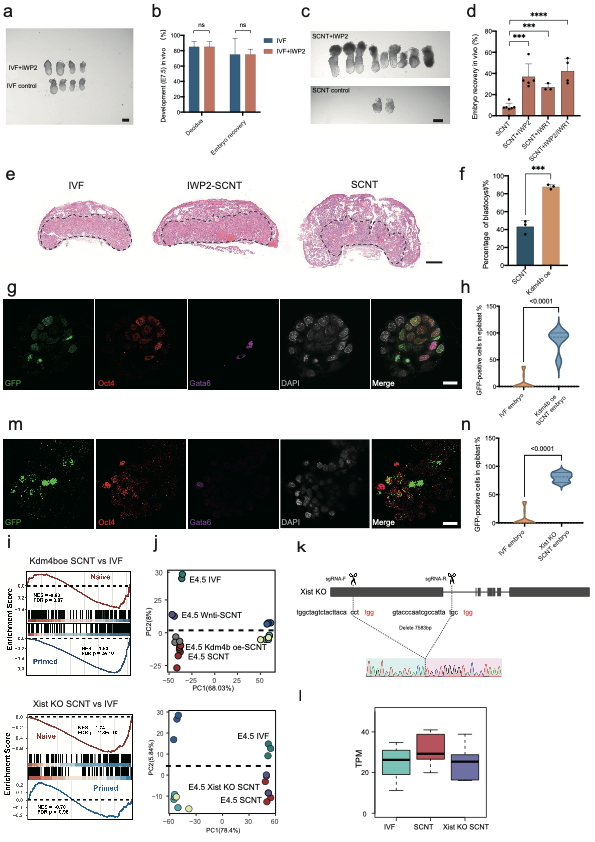


**Supplementary Figure 7. Molecular defects of EPI in *Kdm4b*oe-SCNT and *Xist* KO embryos at the peri-implantation stage.**

(a) Bright-field images of the Wnti treated IVF embryos recovered at E7.5. Top: IWP2 treated IVF embryos. Bottom: untreated IVF embryos. Scale bar: 200 µm.

(b) Percentage of in vivo implantation and embryo recovery at E7.5 in the untreated and IWP2 treated IVF embryos.

(c) Bright-field images of the Wnti treated SCNT embryos recovered at E7.5. Top: IWP2 treated SCNT embryos. Bottom: untreated SCNT embryos. Scale bar: 200 µm.

(d) Percentage of in vivo embryo recovery at E7.5 in untreated and Wnti treated SCNT embryos.

(e) Representative images of histological sections of E19.5 placentae stained with hemotoxylin-eosin (HE). Scale bar: 1000 µm.

(f) Percentage of SCNT embryos reached blastocyst stage following *Kdm4b* injection.

(g, m) TCF/Lef:H2B-GFP *Kdm4b* injection (g) and *Xist* KO (l) SCNT embryos at E5.0 stained for *Oct4*, *Gata6* and DAPI. Scale bar, 20 µm.

(h, n) Percentage of GFP-positive cells in the EPI of IVF and *Kdm4b* injection SCNT embryos (h) and *Xist* KO SCNT embryos (n).

(i) GSEA of specific genes expressed in naïve and primed ESCs between late E4.5 EPI of IVF and *Kdm4b*-injection and *Xist KO* SCNT embryos. NES, normalized enrichment score.

(j) PCA comparison of gene expression profiles among late E4.5 EPI of IVF-, SCNT-, Kdm4b oe- (upper), *Xist* KO (down) SCNT embryos, naïve ESCs, primed ESCs and RSC

(k) Schematic representation of the 7.5 Kb *Xist* deletion and Sanger sequencing results.

(l) RNA-seq analysis on the expression of *Xist* between IVF, SCNT and *Xist* KO SCNT embryos. error bars and means ± SD shown for *n* = 3 biological replicates.

For (b), (d) and (f), *p*-values were determined using unpaired two-tailed *t*-test; error bars and means ± SD shown for *n* ≥ 3 biological replicates.

**Supplementary Figure 8. Characterization of cellular heterogeneity of IVF and SCNT embryos.**

(a) UMAP visulization of scRNA-seq cells of IVF and SCNT embryos at the E2.5, E3.5 and late E4.5 stage.

(b) Cluster information of scRNA-seq analysis identified by shared nearest neighbor (SNN) algorithm in Seurat package.

(c) Normalized expression levels for representative lineage-specific markers.

(d) Heatmap of representative DEGs identified by comparison with the cells derived from IVF embryos at E2.5, E3.5 and late E4.5 stage, for late E4.5 stage, the cells are grouped into EPI, PrE and ExE lineage based on the cluster information deduced by SNN method.

(e) Projection of lineage marker expression levels on the pseudotime trajectory: *Sox2* (EPI), *Sox17* (PrE) and *Eomes* (ExE).

(f) Comparison of naive and primed markers expression between IVF, SCNT, Wnti-SCNT cells at late E4.5, error bars indicate the standard error of the mean (SE) by calculating on the normalized expression for each type of embryo.

| **Groups** | **Donors** | **No. two-cell embryos (% of cultured)** | **No. blastocysts (% of cleaved)** | **No. of blastocysts transferred (No. recipients)** | **No. of deciduas at E7.5 (% of transferred)** | **No. Embryo recovered at E7.5 (% of transferred)** | **No. of cloned offsprings (% of transferred)** | **Bodyweight at birth (g ± SD)** | **Placental weight at birth (g ± SD)** |
| --- | --- | --- | --- | --- | --- | --- | --- | --- | --- |
| Control | BDF1 Cumulus | 252 (88.1) | 118 (46.8) | 94 (7) | 69 (73.4) | 7 (7.45) |  |  |  |
|  | BDF1 Cumulus | 356 (91.0) | 171 (48.0) | 138 (10) |  |  | 1 (0.72) | 1.41 | 0.3 |
|  | BDF1 Sertoli | 375 (71.0) | 215 (57.3) | 167 (9) |  |  | 0 (0) | - | - |
|  | *Xist* KO Sertoli | 291 (73.9) | 199 (68.4) | 107 (8) |  |  | 3 (2.80) | 1.42±0.34 | 0.29±0.03 |
| IWP2 | BDF1 Cumulus | 302 (83.2) | 126 (41.7) | 67 (5) | 56 (83.6) | 24 (35.82) |  |  |  |
|  | BDF1 Cumulus | 186 (91.2) | 90 (48.4) | 71 (6) |  |  | 5 (7.04) | 1.24±0.19 | 0.24±0.05 |
| IWR1 | BDF1 Cumulus | 131 (85.6) | 59 (45.0) | 41 (3) | 33 (80.5) | 11 (26.83) |  |  |  |
|  | BDF1 Cumulus | 234 (89.3) | 98 (41.8) | 46 (3) |  |  | 3 (6.52) | 1.25±0.13 | 0.26±0.04 |
| IWP2+IWR1 | BDF1 Cumulus | 175 (81.4) | 82 (46.9%) | 54 (4) | 49 (90.7) | 22 (40.74) |  |  |  |
|  | BDF1 Cumulus | 205 (91.1) | 78 (38%) | 54 (4) |  |  | 3 (5.55) | 1.29±0.03 | 0.22±0.07 |
| IWP2 | BDF1 Sertoli | 102 (78.5) | 65 (63.7) | 39 (5) |  |  | 4 (10.26) | 1.24±0.08 | 0.22±0.04 |
| IWP2+IWR1 | BDF1 Sertoli | 119 (81.5) | 71 (59.7) | 40 (4) |  |  | 2 (5.00) | 1.4±0.04 | 0.24±0.01 |
| *Kdm4b*oe+si*Dnmt3a/b* | BDF1 Cumulus | 107 (95.5) | 100 (93.5) | 55 (5) |  |  | 5 (9.09) | 1.25±0.11 | 0.21±0.04 |
| *Kdm4b*oe+si*Dnmt3a/b*+IWR1 | BDF1 Cumulus | 71 (92.2) | 62 (87.3) | 40 (4) |  |  | 7 (17.50) | 1.3±0.11 | 0.24±0.03 |
| *Kdm4b*oe+si*Dnmt3a/b*+IWP2+IWR1 | BDF1 Cumulus | 81 (95.3) | 72 (88.9) | 45 (4) |  |  | 7 (15.56) | 1.36±0.16 | 0.21±0.04 |
| IWP2 | *Xist* KO Sertoli | 161 (75.2) | 96 (60.0) | 75 (6) |  |  | 6 (8.00) | 1.32±0.11 | 0.15±0.03 |
| IWP2+IWR1 | *Xist* KO Sertoli | 168 (82.4) | 102 (60.7) | 72 (6) |  |  | 9 (12.50) | 1.24±0.08 | 0.17±0.04 |
| si*Dnmt3a/b*+IWP2 | *Xist* KO Sertoli | 304 (71) | 206 (67.8) | 77 (8) |  |  | 16 (20.78) | 1.32±0.21 | 0.17±0.03 |

**SUPPLEMENTARY TABLES**

**Supplementary Table 1. Developmental rate of SCNT embryos.**

**REFERANCES**

1. Gao, R., et al., *Inhibition of Aberrant DNA Re-methylation Improves Post-implantation Development of Somatic Cell Nuclear Transfer Embryos.* Cell Stem Cell, 2018. **23**(3): p. 426-435 e5.

2. Picelli, S., et al., *Full-length RNA-seq from single cells using Smart-seq2.* Nat Protoc, 2014. **9**(1): p. 171-81.

3. Brind'Amour, J., et al., *An ultra-low-input native ChIP-seq protocol for genome-wide profiling of rare cell populations.* Nat Commun, 2015. **6**: p. 6033.

4. Wang, C., et al., *Reprogramming of H3K9me3-dependent heterochromatin during mammalian embryo development.* Nat Cell Biol, 2018. **20**(5): p. 620-631.

5. Liu, X., et al., *Distinct features of H3K4me3 and H3K27me3 chromatin domains in pre-implantation embryos.* Nature, 2016. **537**(7621): p. 558-562.

6. Gao, S., et al., *Publisher Correction: Tracing the temporal-spatial transcriptome landscapes of the human fetal digestive tract using single-cell RNA-sequencing.* Nat Cell Biol, 2018. **20**(10): p. 1227.

7. Li, L., et al., *Single-Cell RNA-Seq Analysis Maps Development of Human Germline Cells and Gonadal Niche Interactions.* Cell Stem Cell, 2017. **20**(6): p. 858-873.e4.
