# Supplementary figures and images for: Inhibition of Wnt activity improves peri-implantation development of somatic cell nuclear transfer embryos
Source: Natl Sci Rev. 2023 Aug 16;10(9):nwad173. doi: 10.1093/nsr/nwad173 (PMC10430793; doi:10.1093/nsr/nwad173)

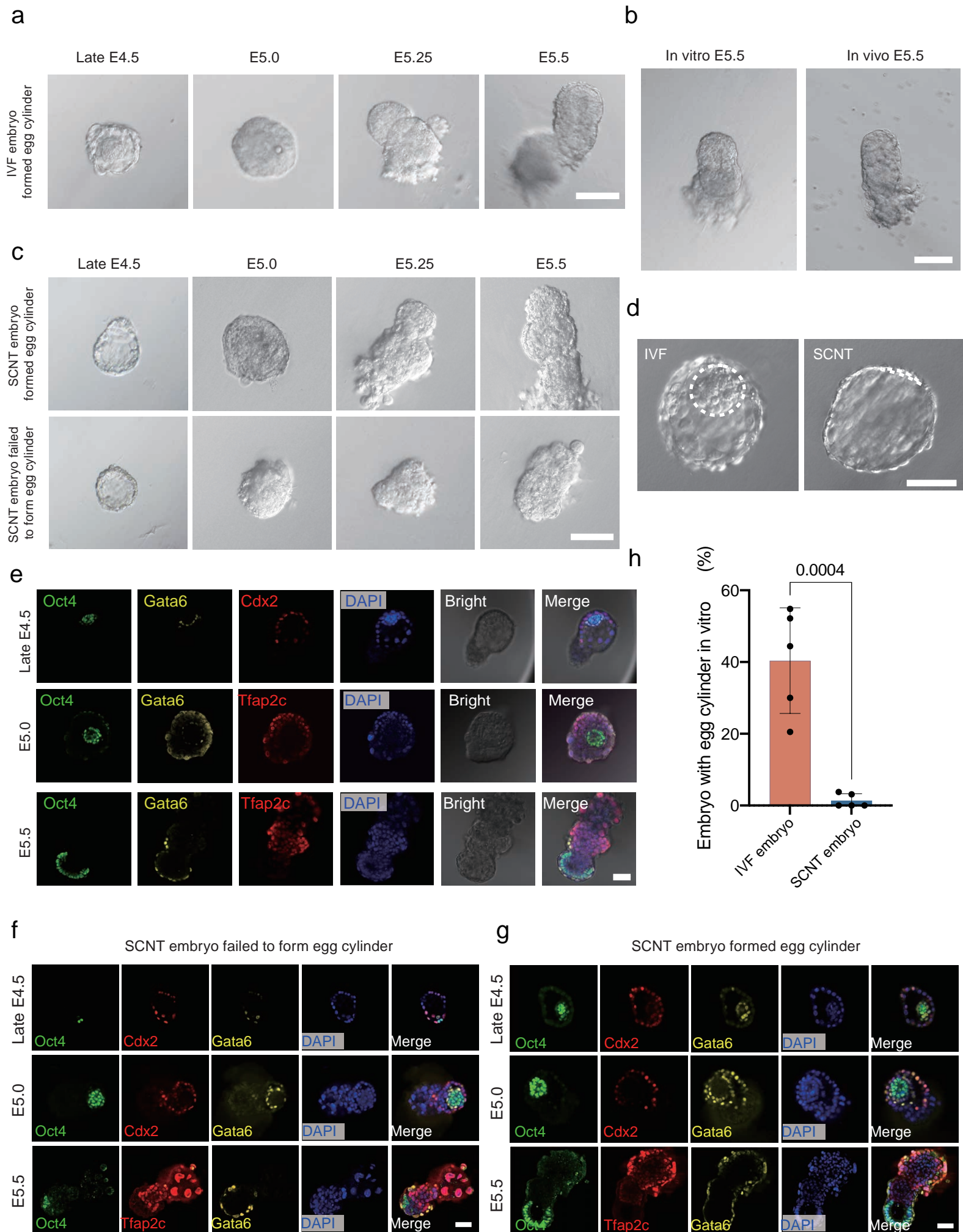

Supplement: nwad173_Supplemental_Files [file nwad173_supplemental_files.zip › Fig. S1.pdf]

a

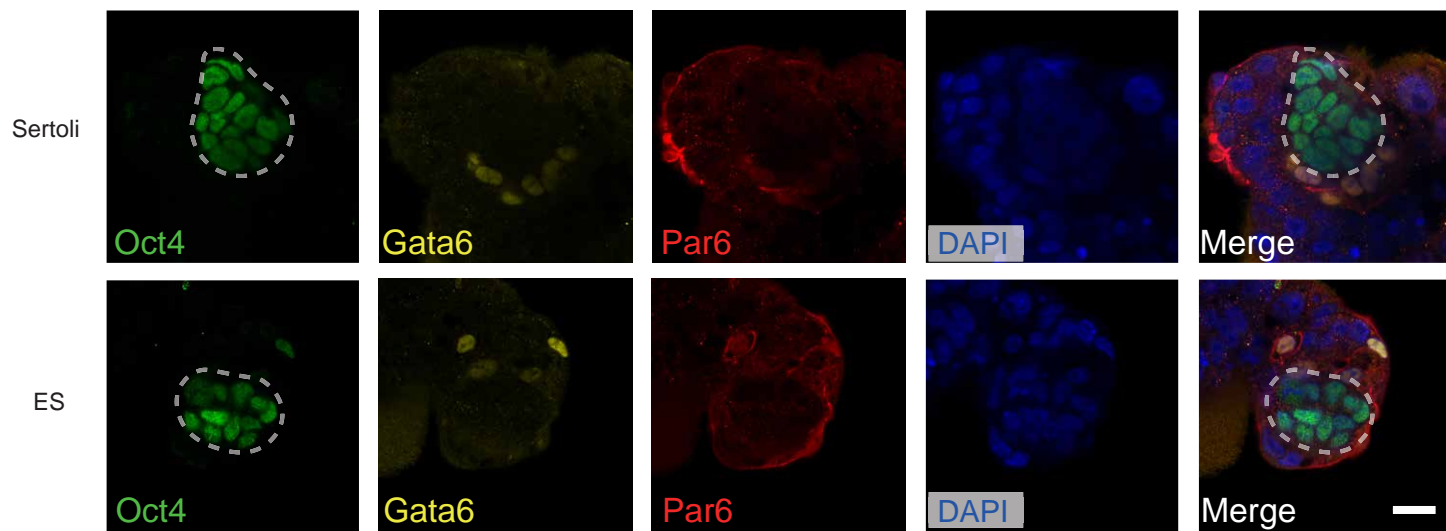

b

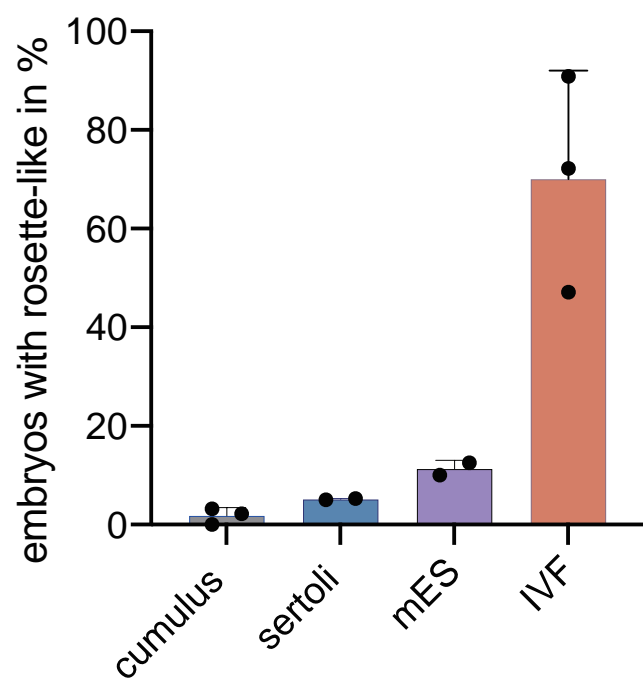

c

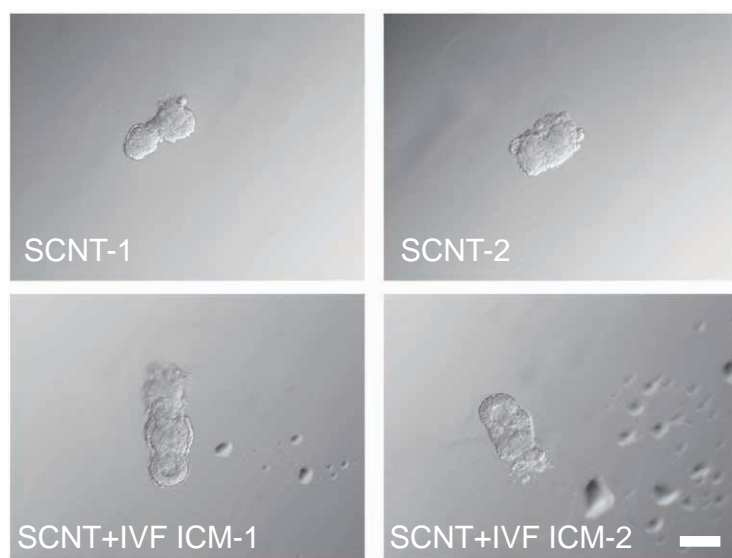

d

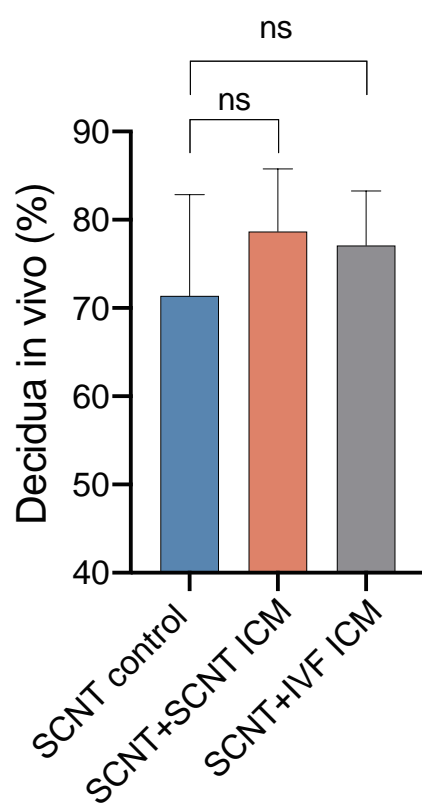

e

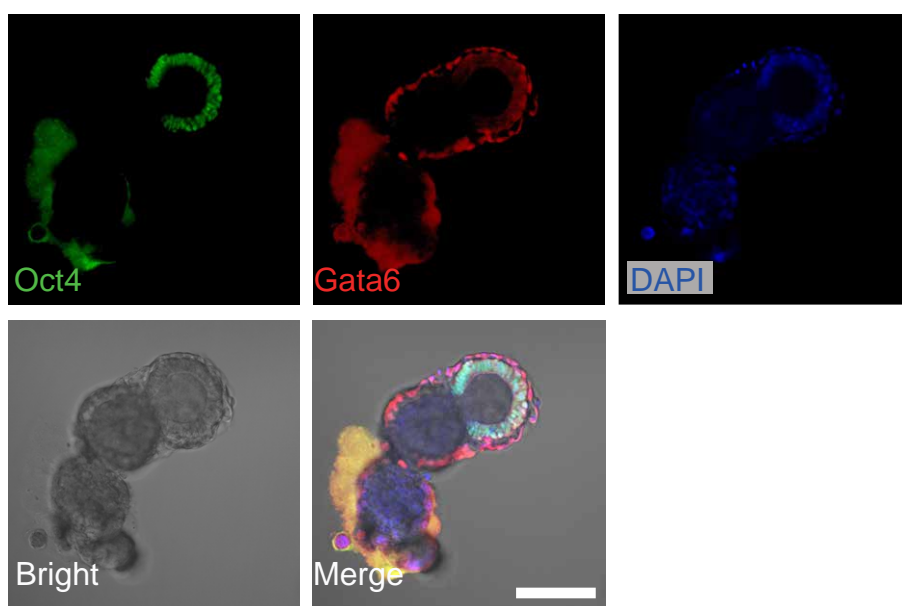

Supplement: nwad173_Supplemental_Files [file nwad173_supplemental_files.zip › Fig. S2.pdf]

a

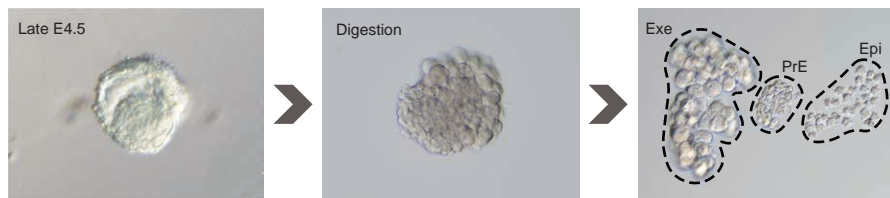

b

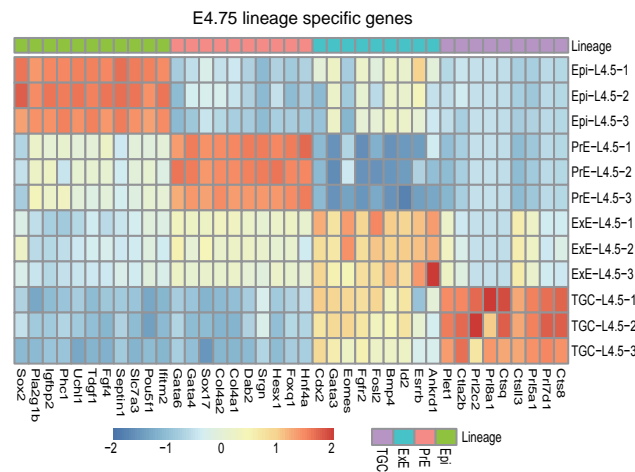

c

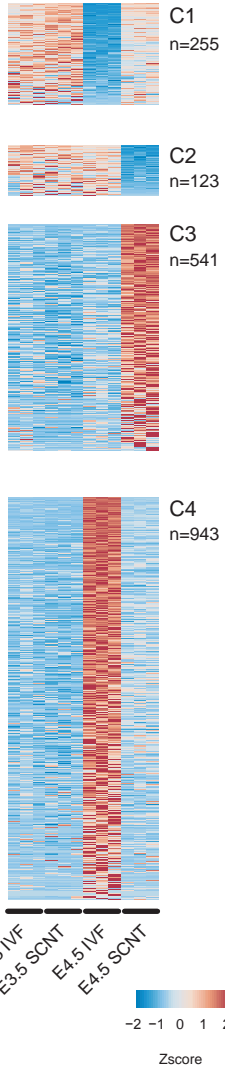

d

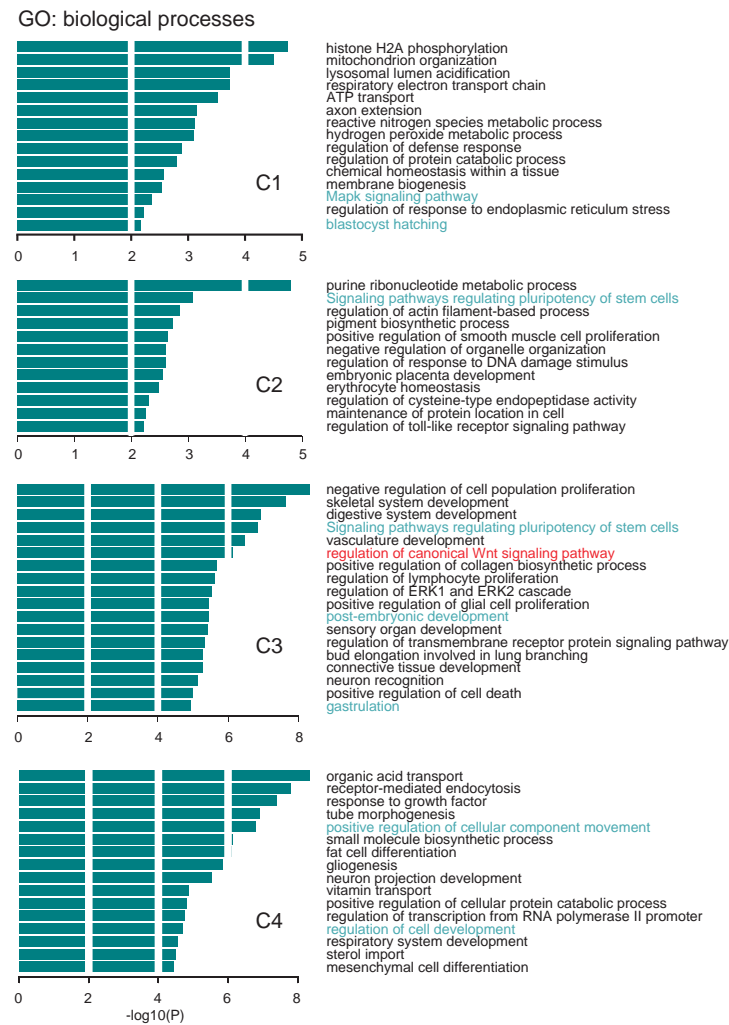

e

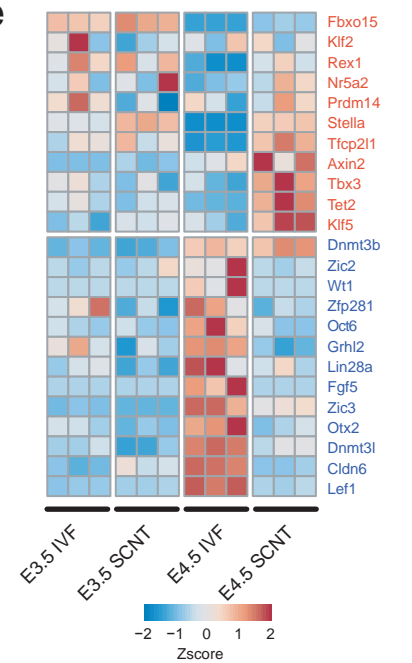

f

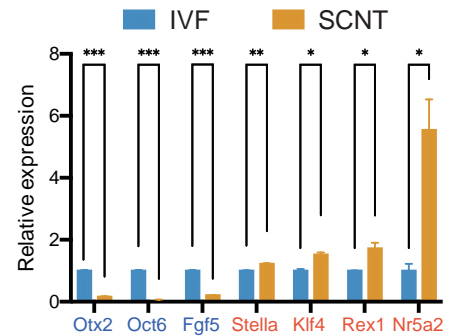

g

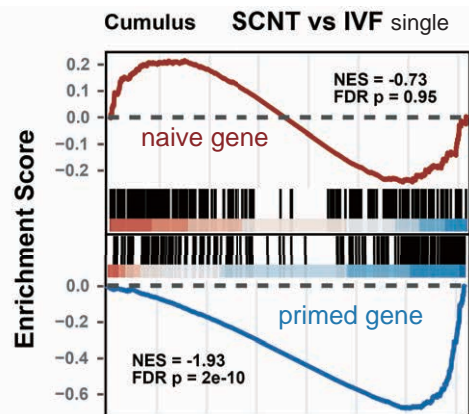

h

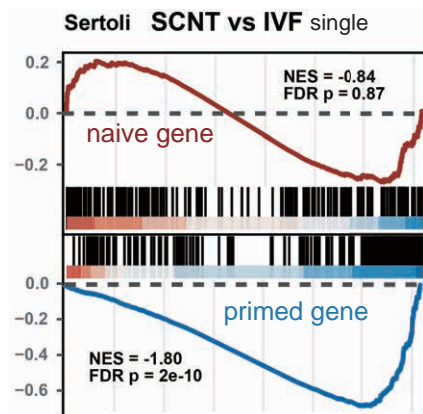

i

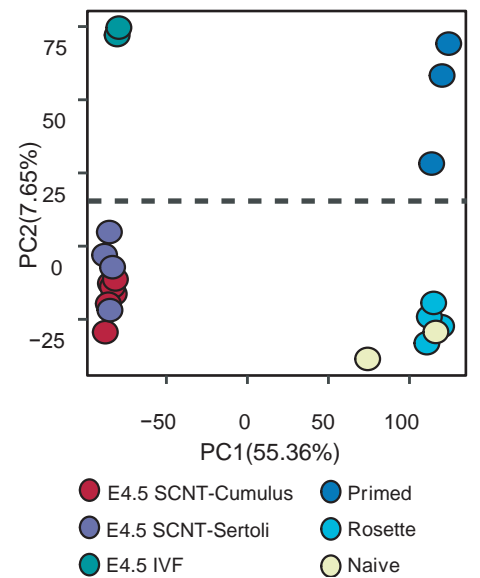

Supplement: nwad173_Supplemental_Files [file nwad173_supplemental_files.zip › Fig. S3.pdf]

a

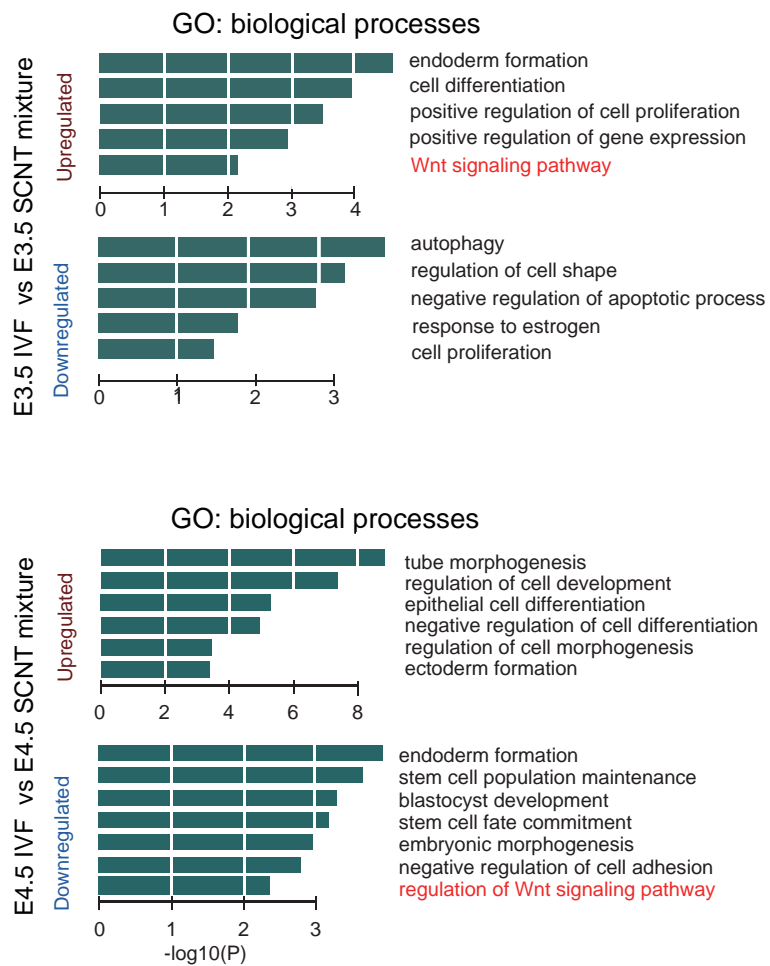

b

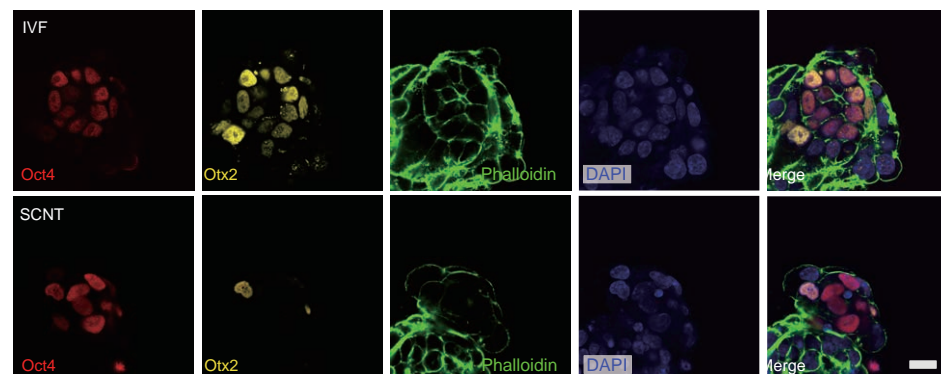

c

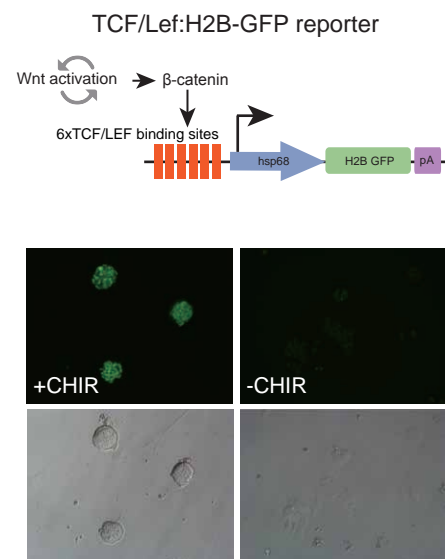

d

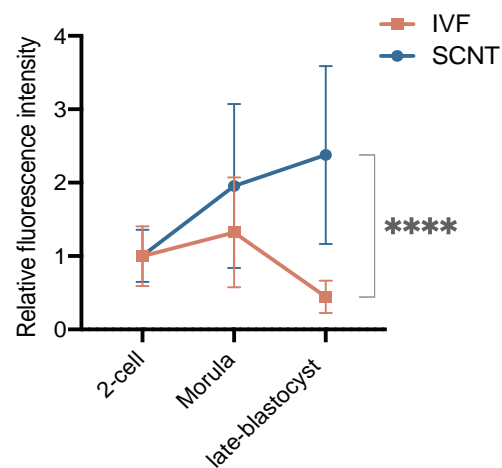

e

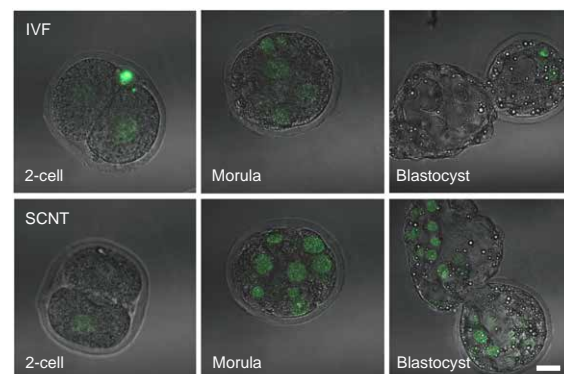

Supplement: nwad173_Supplemental_Files [file nwad173_supplemental_files.zip › Fig. S5.pdf]

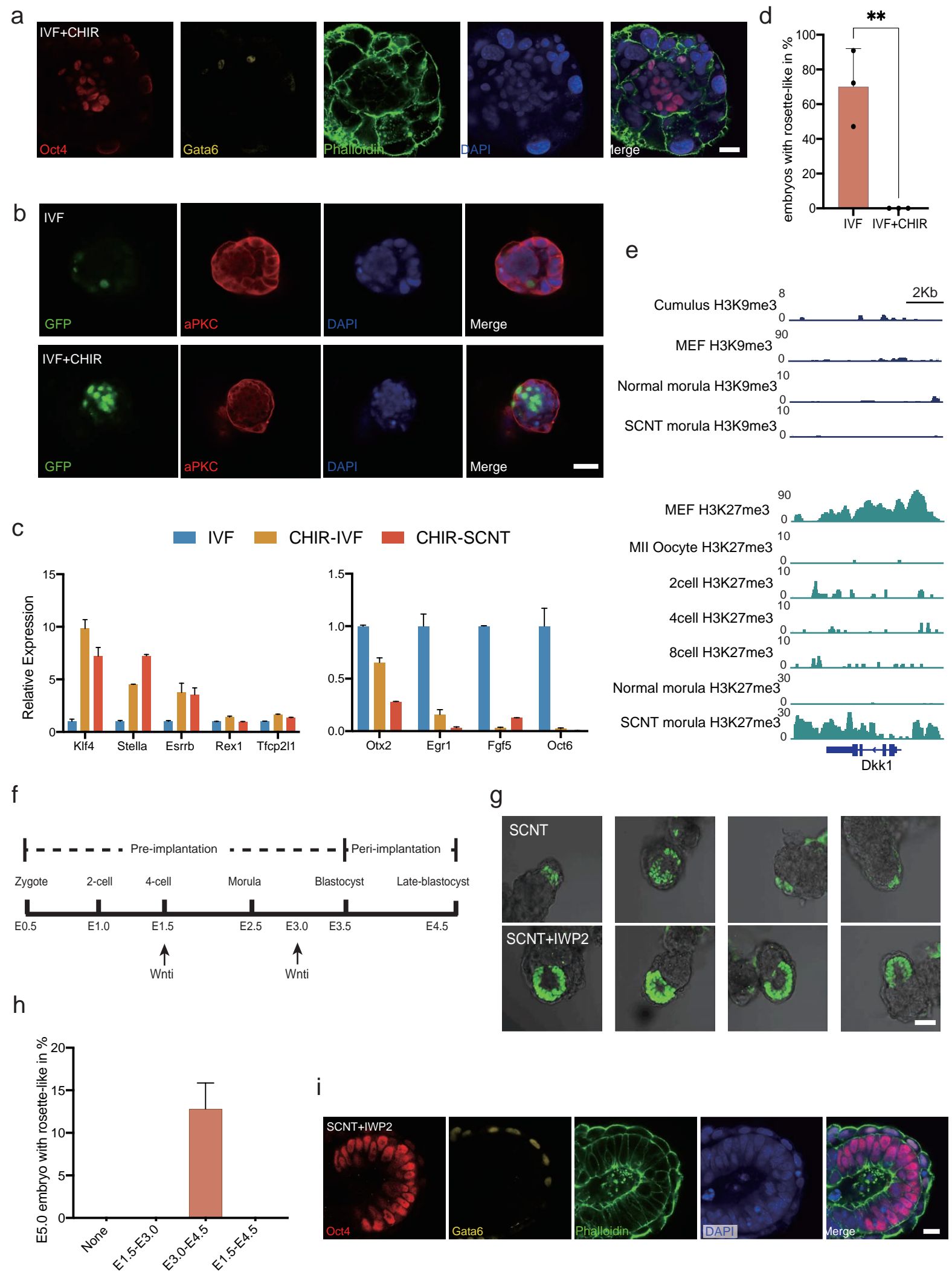

Supplement: nwad173_Supplemental_Files [file nwad173_supplemental_files.zip › Fig. S6.pdf]

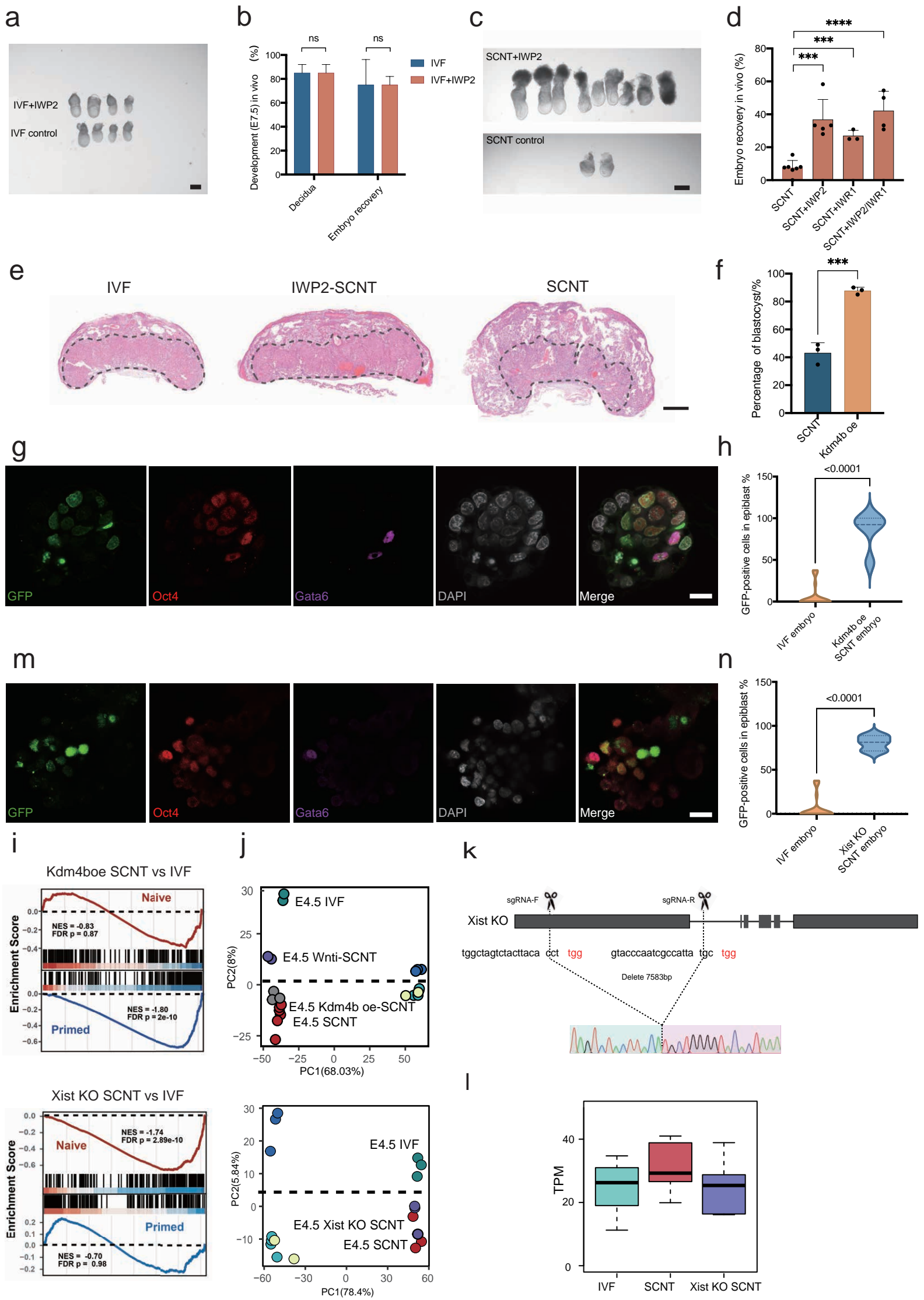

Supplement: nwad173_Supplemental_Files [file nwad173_supplemental_files.zip › Fig. S7.pdf]

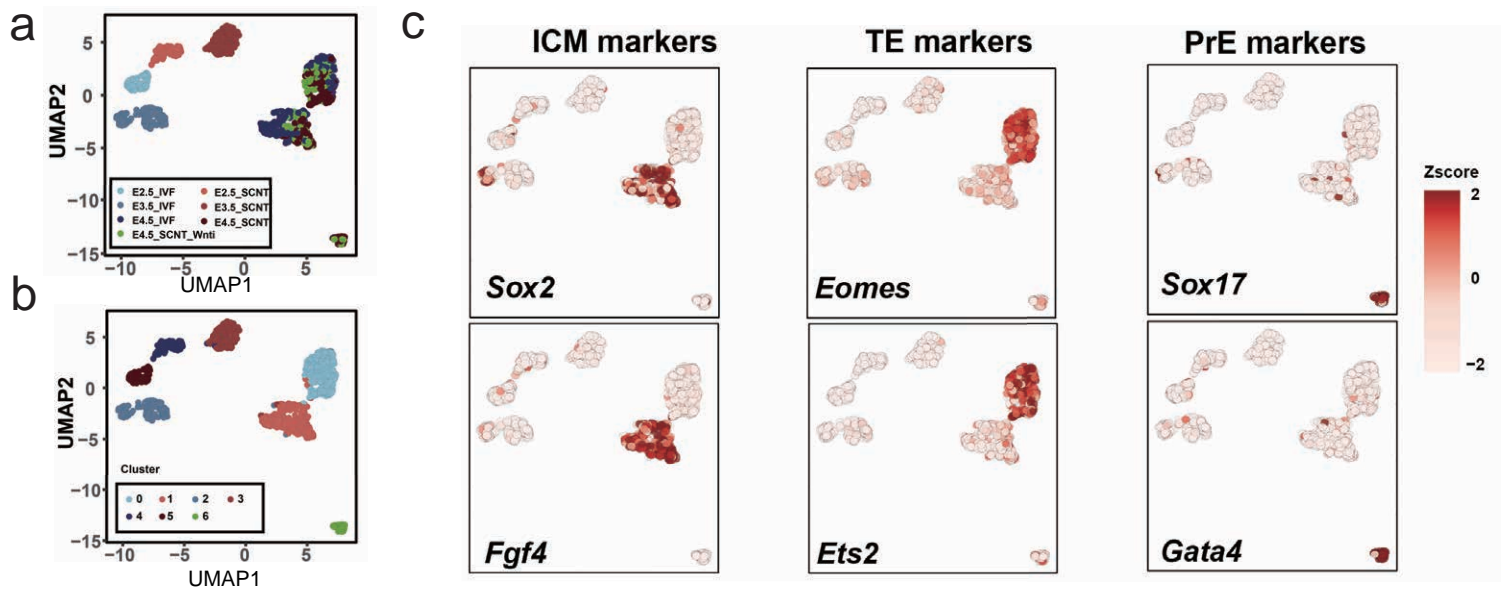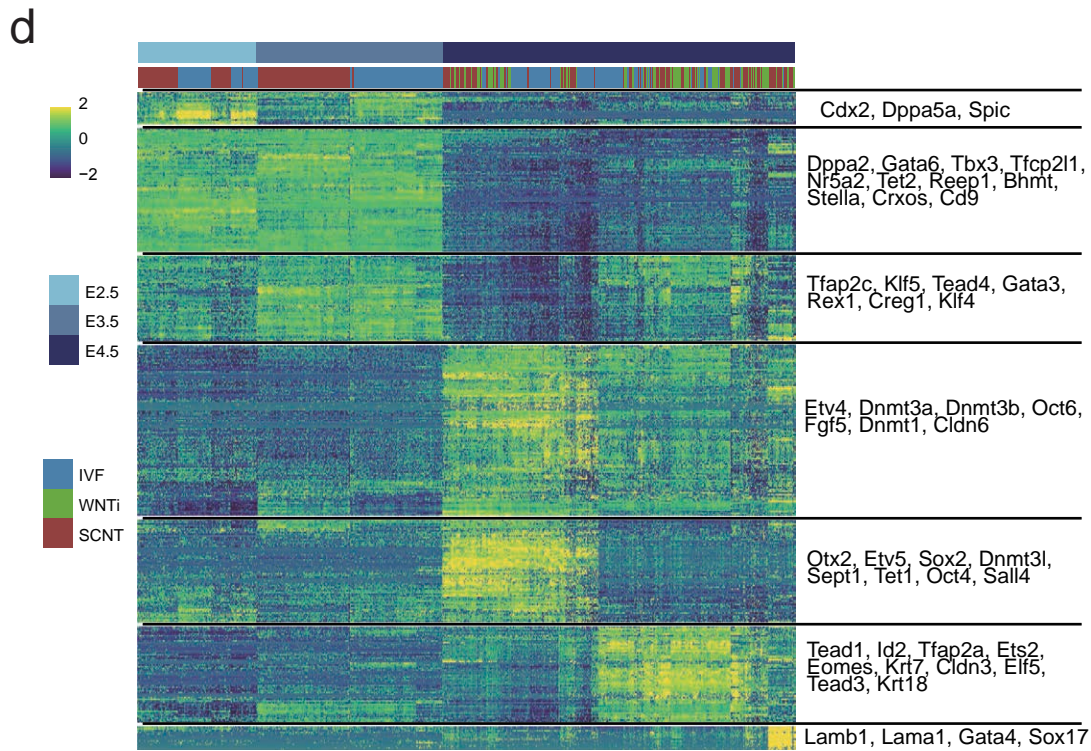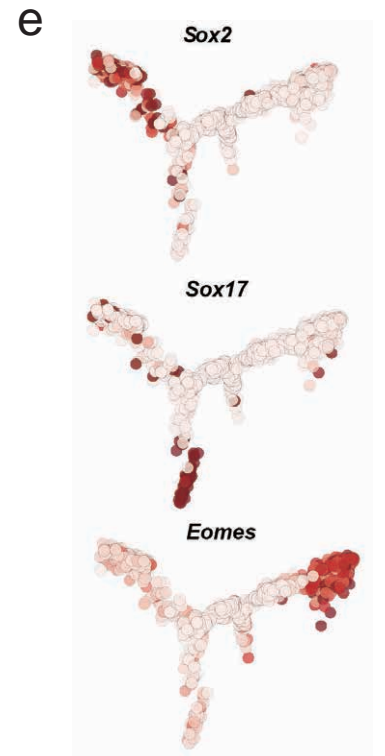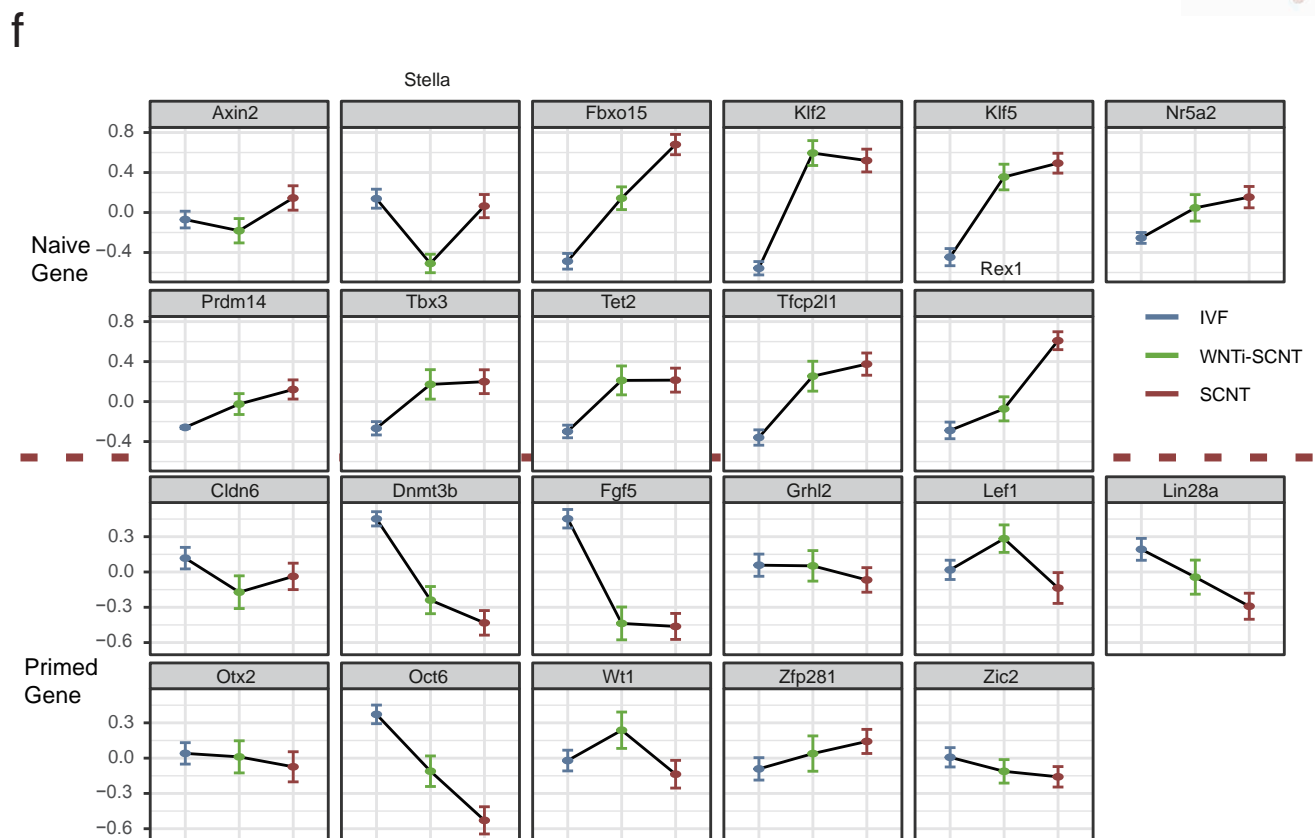

Supplement: nwad173_Supplemental_Files [file nwad173_supplemental_files.zip › Fig. S8.pdf]
