# Supplementary material for: Inhibition of Wnt activity improves peri-implantation development of somatic cell nuclear transfer embryos
Source: Natl Sci Rev. 2023 Aug 16;10(9):nwad173. doi: 10.1093/nsr/nwad173 (PMC10430793; doi:10.1093/nsr/nwad173)
Supplement: nwad173_Supplemental_Files [file Fig. S4.pdf › Fig. S4.pdf]

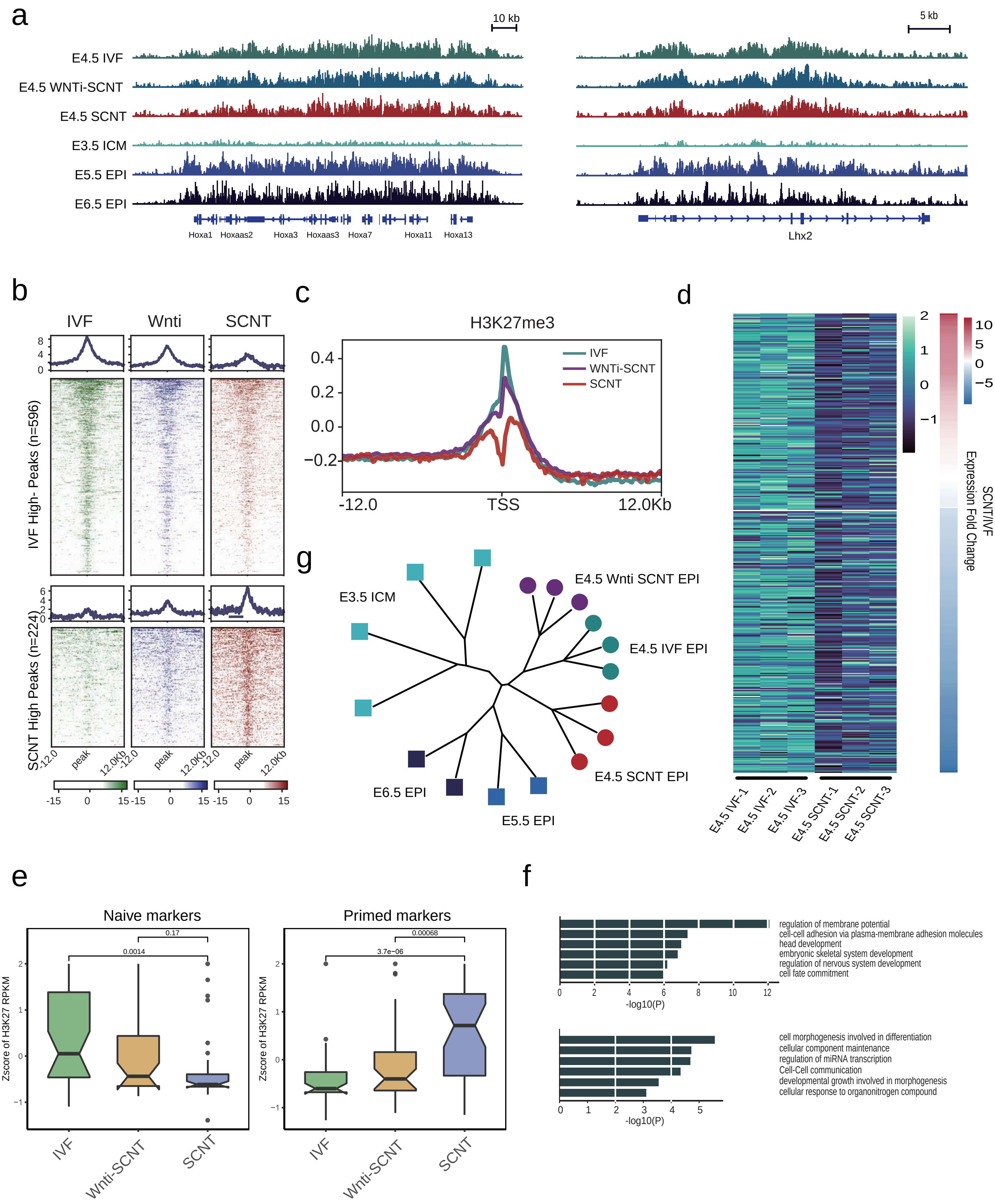

a
10 kb
5 kb
E4.5 IVF
E4.5 WNTi-SCNT
E4.5 SCNT
E3.5 ICM
E5.5 EPI
E6.5 EPI
Hoxa1
Hoxaas2
Hoxa3
Hoxaas3
Hoxa7
Hoxa11
Hoxa13
Lhx2
b
IVF
Wnti
SCNT
IVF High- Peaks (n=596)
SCNT High Peaks (n=224)
-12.0
peak
12.0Kb
-15
0
15
c
H3K27me3
IVF
WNTi-SCNT
SCNT
0.4
0.2
0.0
−0.2
-12.0
TSS
12.0Kb
d
2
1
0
−1
10
5
0
−5
SCNT/IVF
Expression Fold Change
E4.5 IVF-1
E4.5 IVF-2
E4.5 IVF-3
E4.5 SCNT-1
E4.5 SCNT-2
E4.5 SCNT-3
g
E3.5 ICM
E4.5 Wnti SCNT EPI
E4.5 IVF EPI
E4.5 SCNT EPI
E6.5 EPI
E5.5 EPI
e
Naive markers
Primed markers
0.17
0.0014
0.00068
3.7e−06
Zscore of H3K27 RPKM
IVF
Wnti-SCNT
SCNT
f
regulation of membrane potential
cell-cell adhesion via plasma-membrane adhesion molecules
head development
embryonic skeletal system development
regulation of nervous system development
cell fate commitment
-log10(P)
cell morphogenesis involved in differentiation
cellular component maintenance
regulation of miRNA transcription
Cell-Cell communication
developmental growth involved in morphogenesis
cellular response to organonitrogen compound
-log10(P)
